# Multidimensional Factors and Predictors of Vulnerability and Frailty in Older Adults: A Systematic Review

**DOI:** 10.3390/healthcare14203370

**Published:** 2026-10-09

**Authors:** Helder Pino, Ana Ramos, Cesar Fonseca, Elisabete Alves, Anabela Coelho

**Affiliations:** 1 Comprehensive Health Research Centre (CHRC), Universidade de Évora, 7000-801 Évora, Portugal; anaramos@enfermagem.ulisboa.pt (A.R.); cfonseca@uevora.pt (C.F.); elisabete.alves@uevora.pt (E.A.); anabela.coelho@uevora.pt (A.C.); 2 Unidade Local de Saúde do Algarve (ULS Algarve), 8000-386 Faro, Portugal; 3 Nursing Research Innovation and Development Centre of Lisbon (CIDNUR), Escola Superior de Enfermagem da Universidade de Lisboa, 1600-096 Lisbon, Portugal; 4 Escola Superior de Enfermagem São João de Deus, Universidade de Évora, 7000-811 Évora, Portugal; 5 Global Health and Tropical Medicine, Instituto de Higiene e Medicina Tropical, Universidade NOVA de Lisboa, 1099-085 Lisbon, Portugal

**Keywords:** frailty, vulnerability, predictors, elderly, geriatric assessment, systematic review

## Abstract

**Highlights:**

**What are the main findings?**

- Mapped candidate vulnerability determinants across five interrelated biopsychosocial and self-care domains.
- Addressed predictor–outcome construct overlap (incorporation bias) to distinguish operational diagnostic criteria from independent prognostic drivers.

**What are the implications of the main findings?**

- Identified candidate variables to inform future predictive modeling and risk screening.

**Abstract:**

Background: Population ageing is characterized by rising multimorbidity and multifaceted vulnerability. While vulnerability is inherently a biopsychosocial state, clinical research predominantly operationalizes risk through physical frailty phenotypes, constraining holistic geriatric care. Identifying the interplay across biological, functional, and psychosocial domains is essential for comprehensive risk evaluation. Objectives: To synthesize multidimensional factors associated with vulnerability and frailty in adults aged ≥65, categorizing cross-sectional correlates and prospective longitudinal predictors to inform multidimensional assessment. Methods: Following PRISMA 2020 guidelines, systematic searches were conducted across PubMed, CINAHL, Scopus, Web of Science, and Google Scholar (2020–2025). Independent reviewers performed screening, selection, extraction, and Joanna Briggs Institute (JBI) critical appraisal (PROSPERO: CRD420251039796). Results: Forty studies (22 cross-sectional, 17 cohort, 1 quasi-experimental) comprising 77,229 cumulative participant observations across community, outpatient, inpatient, and residential care settings were synthesized. Thirty-seven candidate determinants were mapped across five domains: biological/clinical, psychological, socioeconomic/relational, lifestyle, and self-care profile. Advanced age, multimorbidity, and polypharmacy were the most frequent clinical factors; prospective cohorts specifically linked high anticholinergic burden and functional dependence in complex activities to incident frailty. Depressive symptoms, cognitive decline, low education, financial strain, and social isolation consistently delineated vulnerable states. Conclusions: Vulnerability in older adults is a multifaceted construct requiring assessment tools that extend beyond isolated physical markers. Rather than a definitive feature set, these identified factors provide an evidence-based pool of candidate variables that may inform future risk-stratification and predictive modeling initiatives, supporting integrated, person-centered care.

## 1. Introduction

Global demographic shifts indicate that by 2050, one in six people worldwide will be aged 65 or older, surpassing 2 billion, an unprecedented demographic transition reaching critical thresholds in more developed regions [1]. However, increased longevity has not translated into proportional improvements in health spans; instead, ageing populations face a rising burden of multimorbidity, chronic disease, and complex health demands [2]. Addressing this shift requires healthcare systems and social policies to transition from fragmented disease management toward integrated, person-centered models of care [1].

Central to this transition is the conceptual differentiation among related geriatric constructs: intrinsic capacity, frailty, functional disability, and vulnerability. In this review, vulnerability is conceptualized as the overarching, dynamic biopsychosocial state of susceptibility to adverse health-related outcomes, shaped by the cumulative interaction of biological, psychological, socio-economic, and environmental determinants over the life course [3,4,5,6]. Within this hierarchy, intrinsic capacity represents the composite of an individual’s physical and mental reserves [7], whereas frailty denotes a specific biological syndrome characterized by cumulative physiological decline and diminished resistance to stressors [8]. In clinical research, frailty and clinical-functional disability are frequently operationalized as clinical proxies or manifestations of this broader vulnerability state [9,10,11]. Establishing this distinction is vital: while primary studies often focus narrowly on physical frailty scales, a comprehensive understanding of vulnerability demands an evaluation across all underlying domains.

Despite this multidimensional conception, existing clinical practice and epidemiological research remain disproportionately anchored in physical frailty phenotypes [9,10]. Yet, vulnerability extends far beyond biological parameters. Susceptibility to decline is heavily driven by social determinants, including lower educational attainment, financial strain, and reduced social engagement [7,11]. As highlighted by OECD data, high out-of-pocket costs for long-term care place up to 95% of care-dependent older adults at risk of poverty, illustrating how economic precarity directly compounds clinical vulnerability [12]. Conversely, robust family networks and psychosocial resources serve as vital protective factors [13].

Functionality and self-care capacities are inextricably linked to this continuum. Unmet care needs often accelerate functional decline, transitioning individuals from independence into states of severe vulnerability [14,15,16]. Self-care—defined as the intrinsic ability of individuals, families, and communities to promote health, manage illness, and maintain autonomy [17]—encompasses cognitive, physical, psychosocial, demographic, and sociocultural dimensions [18,19,20]. Preserving functional autonomy and self-care performance represents a critical barrier against adverse events such as falls, preventable hospitalizations, and institutionalization [8,21,22].

Currently, there is no universally accepted gold standard for screening or quantifying multidimensional vulnerability, resulting in substantial variation across healthcare settings and scientific disciplines [5,23]. While several systematic reviews have examined predictors of physical frailty [24,25], there is a marked scarcity of syntheses examining the comprehensive spectrum of non-biological, social, and functional factors that underpin global vulnerability across both community and acute healthcare contexts. Moreover, the integration of electronic health records and emergent analytical technologies highlights the need to systematically identify which multi-domain variables warrant clinical prioritization [25].

To address these gaps, this systematic review synthesizes the multidimensional factors associated with vulnerability and frailty in adults aged 65 and older. By examining both cross-sectional correlates and longitudinal predictors across hospital and community settings, this review aims to clarify domain-specific determinants and outline an evidence-based pool of candidate variables to inform comprehensive clinical assessment and future risk-stratification models.

## 2. Materials and Methods

### 2.1. Protocol Registration, Published Protocol, and Operational Refinements

This systematic review was conducted and reported in accordance with the Preferred Reporting Items for Systematic Reviews and Meta-Analyses (PRISMA 2020) guidelines. The review was prospectively registered in PROSPERO on 02 June 2025 (registration number: CRD420251039796; first submitted 31 May 2025), and the foundational study protocol was subsequently published in full [26].

The core research question, target population (≥65 years), primary focus on multidimensional vulnerability/frailty, and the a priori exclusion of acute environmental shocks (COVID-19, natural disasters) strictly adhered to both the registered PROSPERO protocol and the published conference protocol. In accordance with PRISMA 2020 guidelines (Item 24c), minor operational refinements implemented during the analytical execution of the review—specifically: standardizing eligibility reporting strictly to the PECO framework; establishing explicit numerical JBI quality cut-offs (≥80% High, 60–79% Moderate, <60% Low); and performing an inductive narrative thematic synthesis rather than quantitative meta-analysis due to severe operational heterogeneity across frailty definitions—are transparently reported and justified in the subsequent methodology subsections.

### 2.2. Research Question

To guide the search strategy, eligibility screening, and narrative synthesis, the research question was structured using the PECO (Population, Exposure, Comparator, Outcome) framework, recommended for observational and prognostic reviews of epidemiological evidence [27,28].

Conceptually, this review distinguishes between multidimensional vulnerability—an overarching biopsychosocial state of diminished physiological reserve and heightened susceptibility to stressors across biological, psychological, and social domains—and frailty, which predominantly serves in the published empirical literature as an operational clinical proxy measured via physical phenotypes, deficit accumulation indices, or multidimensional assessment scales.

The PECO parameters were operationalized across three interconnected evidence streams:

Population (P): Older adults aged 65 years and older residing in community, acute-care (hospital), or institutional/residential aged care settings.

Exposure (E): Multidimensional candidate determinants and risk factors (biological/clinical, psychological, socioeconomic/relational, behavioral/lifestyle, and self-care profile) and, for downstream prognostic analyses, established baseline vulnerability or frailty states.

Comparator (C): Robust, non-frail, or non-vulnerable reference groups; individuals unexposed to specific risk factors; or lower vulnerability/frailty exposure tiers.

Outcomes (O)**:** Defined according to the epidemiological focus: (i) Concurrent frailty/vulnerability states assessed cross-sectionally via validated instruments; (ii) Longitudinal trajectories, including incident frailty, frailty progression, or bidirectional state transitions; and (iii) Downstream adverse clinical endpoints resulting from established frailty/vulnerability (specifically, hospitalization, institutionalization, and mortality).

Primary Research Questions:

“Which multidimensional factors are cross-sectionally associated with, or prospectively predict, vulnerability and frailty onset and progression in older adults aged ≥65 years across community, hospital, and residential settings?”

“To what extent do established vulnerability and frailty states prospectively predict downstream adverse clinical outcomes (hospitalization, institutionalization, and mortality) in this population?”

### 2.3. Search Strategy

Between 2 and 5 June 2025, a comprehensive and systematic bibliographic search was executed and records were exported across scientific databases: PubMed/MEDLINE (2 June 2025), Scopus (3 June 2025), Web of Science (4 June 2025), and CINAHL (4 June 2025), followed by Google Scholar on 5 June 2025. These execution dates were strictly distinguished from the publication date filters applied to the records: all database searches were restricted to publications from 2020 onwards, with CINAHL and Web of Science applying an exact publication cutoff up to 29 May 2025 (reflecting the date of protocol finalization), while PubMed and Scopus applied standard 2020–2025 annual limits (full search syntax details and interface parameters are provided in Table A1, Appendix A). To achieve a targeted and precise search, we employed a combination of specific keywords and Mesh Terms (Medical Subject Headings), using the keywords “Vulnerable Populations”, “Frail Elderly”, “Functionally Impaired Elderly”, “Geriatric Assessment”, “International Classification of Functioning, Disability and Health”, “Health Status Indicators”, “Quality of Life”, “prognosis” and “predictor” and their variations, opportunely combined by Boolean operators. Additionally, a targeted search was conducted in Google Scholar to retrieve non-indexed grey literature, specifically academic dissertations, theses, and institutional reports. Using an advanced title query (allintitle: ((Vulnerable OR FRAIL OR risk) AND aged 65)), the first 100 consecutive records sorted by default algorithmic relevance were screened, in accordance with established methodological guidance for evidence syntheses [29] (full search syntax details are provided in Table A1, Appendix A). Only articles published in English, French, Portuguese, or Spanish were included.

In the initial screening phase, two expert reviewers (HP and AR) independently assessed all titles and abstracts extracted from the electronic database searches. Using the software (https://new.rayyan.ai/) (accessed on 5 June 2025), duplicate and irrelevant records were systematically eliminated. In instances of discordance, a third expert reviewer (AC) was consulted to facilitate consensus. For studies deemed potentially relevant during initial screening, full-text articles were obtained. Each of these was subjected to a rigorous independent assessment by the reviewers (HP and AR) in alignment with predetermined eligibility criteria. In situations where consensus was challenging, dialogues were initiated between the primary reviewers. If no agreement was reached, the adjudication of the third reviewer (CF), previously uninvolved, was sought to ensure an unbiased decision-making process.

### 2.4. Eligibility Criteria

To ensure methodological transparency and rigor, eligibility criteria were defined a priori using the PECO framework [28], structured into explicit inclusion and exclusion parameters. Inclusion Criteria: Population: Older adults aged ≥65 years residing in community, acute-care, long-term care, or institutional settings. Exposures/Determinants: Studies evaluating at least one multidimensional determinant (e.g., biological, clinical, functional, psychological, cognitive, behavioral, or socioeconomic/relational factors). Outcomes: Vulnerability or frailty assessed using validated clinical instruments, multidimensional indices, or deficit accumulation models. Study Design: Primary quantitative observational and longitudinal empirical studies (prospective or retrospective cohort studies, cross-sectional investigations, and quasi-experimental studies). Language and Period: Peer-reviewed articles published between 2020 and 2025 in English, French, Portuguese, or Spanish. The search was restricted to publications from 2020 onwards to capture the most contemporary epidemiological profiles, reflect recent advancements in multidimensional frailty screening, and align with current clinical practice guidelines.

Exclusion Criteria: Population: Studies focusing exclusively on populations aged <65 years. Furthermore, studies involving mixed-age populations were completely excluded if the data for the older adult cohort (≥65 years) were not disaggregated and reported separately. Operationalization: Studies relying on non-validated screening scales, unstandardized proxy self-reports, or those failing to specify the measured dimension of vulnerability or frailty. Contextual and Stressor Confounders (Acute Disruptions): Studies investigating the transient impact of acute, catastrophic environmental or societal shocks—specifically the SARS-CoV-2 pandemic, natural disasters, or acute armed conflict. While our review conceptualizes vulnerability as encompassing environmental and social contexts, these catastrophic shocks represent acute, non-endemic disruptors that confound baseline, chronic biopsychosocial vulnerability. Excluding acute shock studies isolates persistent, everyday risk profiles relevant to routine clinical practice. Methodological Designs: Secondary syntheses (systematic reviews, meta-analyses), study protocols, commentaries, conference abstracts, and purely psychometric or linguistic validation studies. While studies were required to apply validated instruments to quantify vulnerability, pure psychometric validation studies (e.g., focusing solely on factor structure, test–retest reliability, or translation without analyzing substantive clinical/social predictors or longitudinal outcomes) were excluded.

### 2.5. Evaluation of Risk of Bias and Methodological Quality of Studies

To maintain methodological rigor, two reviewers (H.P. and A.R.) independently assessed the methodological quality and risk of bias of all eligible studies using the official Joanna Briggs Institute (JBI) Critical Appraisal Checklists: the *JBI Critical Appraisal Checklist for Analytical Cross-Sectional Studies* (8 items), the *JBI Critical Appraisal Checklist for Cohort Studies* (11 items), and the *JBI Critical Appraisal Checklist for Quasi-Experimental Studies (non-randomized experimental studies)* (9 items). Discrepancies were resolved through structured consensus discussions, with adjudication by a third senior reviewer (A.C. or C.F.) when required.

Because official Joanna Briggs Institute (JBI) methodological guidance does not prescribe standardized numerical cut-off scores or mandatory exclusion rules [30]—leaving decisions regarding scoring rubrics and threshold criteria to the review team’s discretion—methodological quality was evaluated through a two-step operational refinement to the protocol (as detailed in Section 2.1): 1. Quality Tier Classification: Studies were categorized into explicit quality strata based on the proportion of affirmative (‘yes’) responses relative to applicable criteria: High quality (≥80%), Moderate quality (60–79%), and Low quality (<60%). These tiers were established to weight and contextualize the robustness of narrative findings in the synthesis. 2. Synthesis Eligibility Threshold: To prevent critical reporting deficiencies or high risk of bias from compromising the qualitative synthesis, a minimum retention threshold of ≥60% was defined, establishing that studies categorized as Low quality (<60%) would be excluded from evidence synthesis.

All 40 eligible primary studies achieved scores ≥ 60% (32 classified as High quality and 8 as Moderate quality; no studies scored <60%), allowing the entire eligible corpus to inform the systematic synthesis (Table 1).

### 2.6. Data Extraction and Synthesis

Data extraction was conducted independently and in duplicate by two reviewers (H.P. and A.R.) using a standardized, pre-piloted electronic data extraction form designed in Microsoft Excel. The extraction template was initially piloted on a random sample of five studies (12.5% of the corpus) to calibrate extraction consistency across reviewers. Extracted variables comprised: study details (first author, publication year, country, healthcare setting); study design; sample size and demographic characteristics; operational definitions and validated tools used to measure vulnerability/frailty; investigated predictor domains; and primary statistical findings.

Discrepancies during extraction were resolved through discussion and re-examination of the primary papers, with persistent disagreements settled by a third senior investigator (C.F. or A.C.). When multiple publications arose from the same longitudinal cohort (e.g., SHARE, HRS, ELSA), we cross-referenced sample sizes, follow-up waves, and reported variables to prevent redundant data entry.

Extracted findings were categorized across three distinct epidemiological roles: (i) factors cross-sectionally associated with frailty or vulnerability states; (ii) prospective predictors of frailty onset, progression, or state transitions in longitudinal cohorts; and (iii) frailty or vulnerability as a baseline predictor of subsequent adverse clinical events (e.g., hospitalization, institutionalization, and mortality).

A narrative, inductive thematic synthesis was conducted to group individual variables into overarching descriptive categories and five analytical domains: biological/clinical, self-care/functional profile, psychological, behavioral, and socioeconomic/relational.

## 3. Results

### 3.1. Study Selection

**Figure 1:**
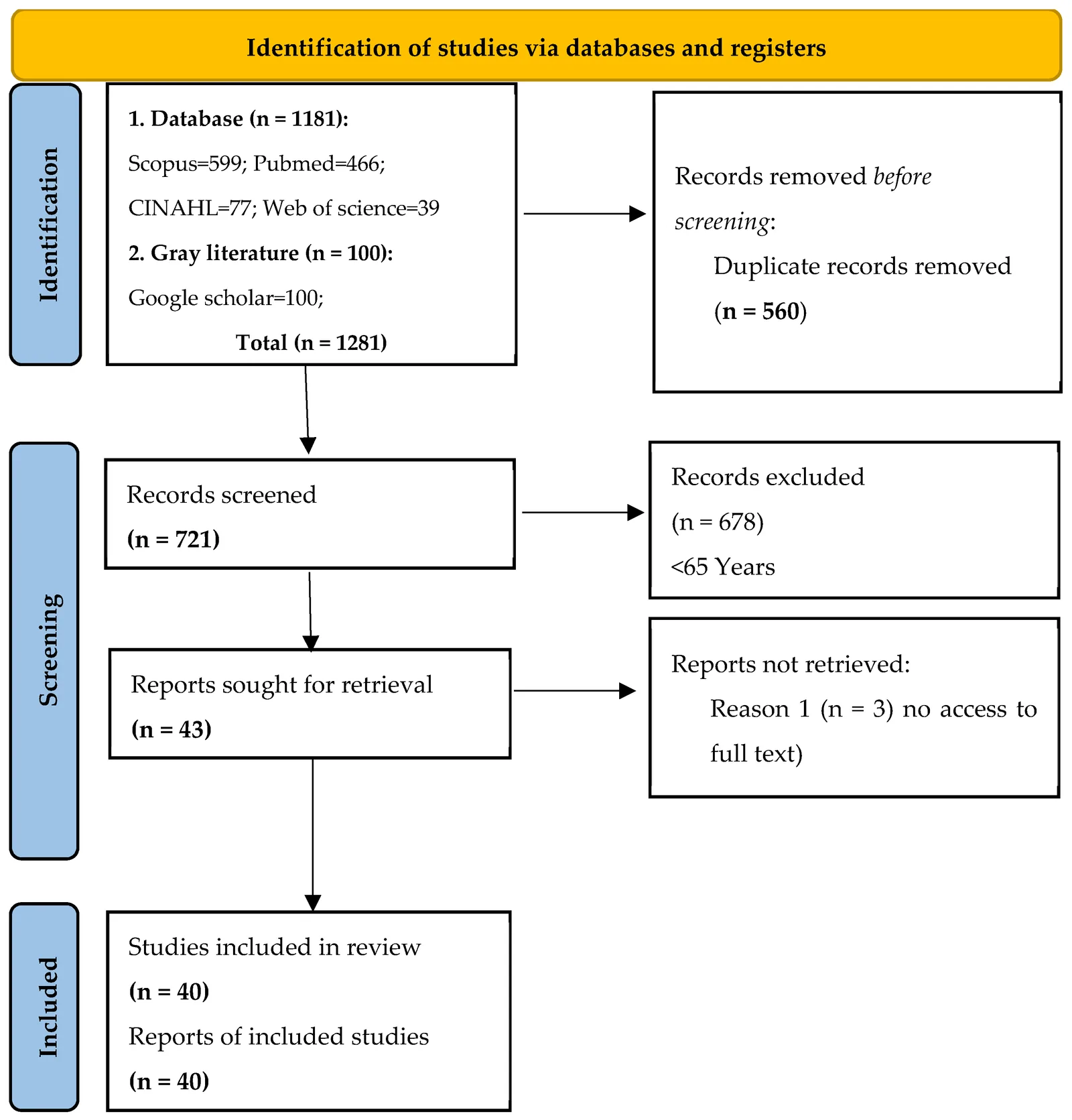
PRISMA flow diagram.

The selection of studies followed a systematic three-stage process: identification, screening, and inclusion. A total of 1281 records were initially identified. This included 1181 records from databases (Scopus: 599; PubMed: 466; CINAHL: 77; Web of Science: 39) and 100 records from grey literature via Google Scholar. Before screening began, 560 duplicate records were removed. The remaining 721 records were screened for relevance. During this phase, 678 records were excluded based on title and abstract, or because the study population was under 65 years of age. This left 43 reports sought for retrieval. Of these, 3 reports could not be retrieved due to a lack of access to the full text. The remaining 40 reports were assessed for eligibility and all met predetermined criteria. Consequently, a total of 40 studies were included in the final systematic review (Figure 1).

### 3.2. Study Characteristics

**Figure 2:**
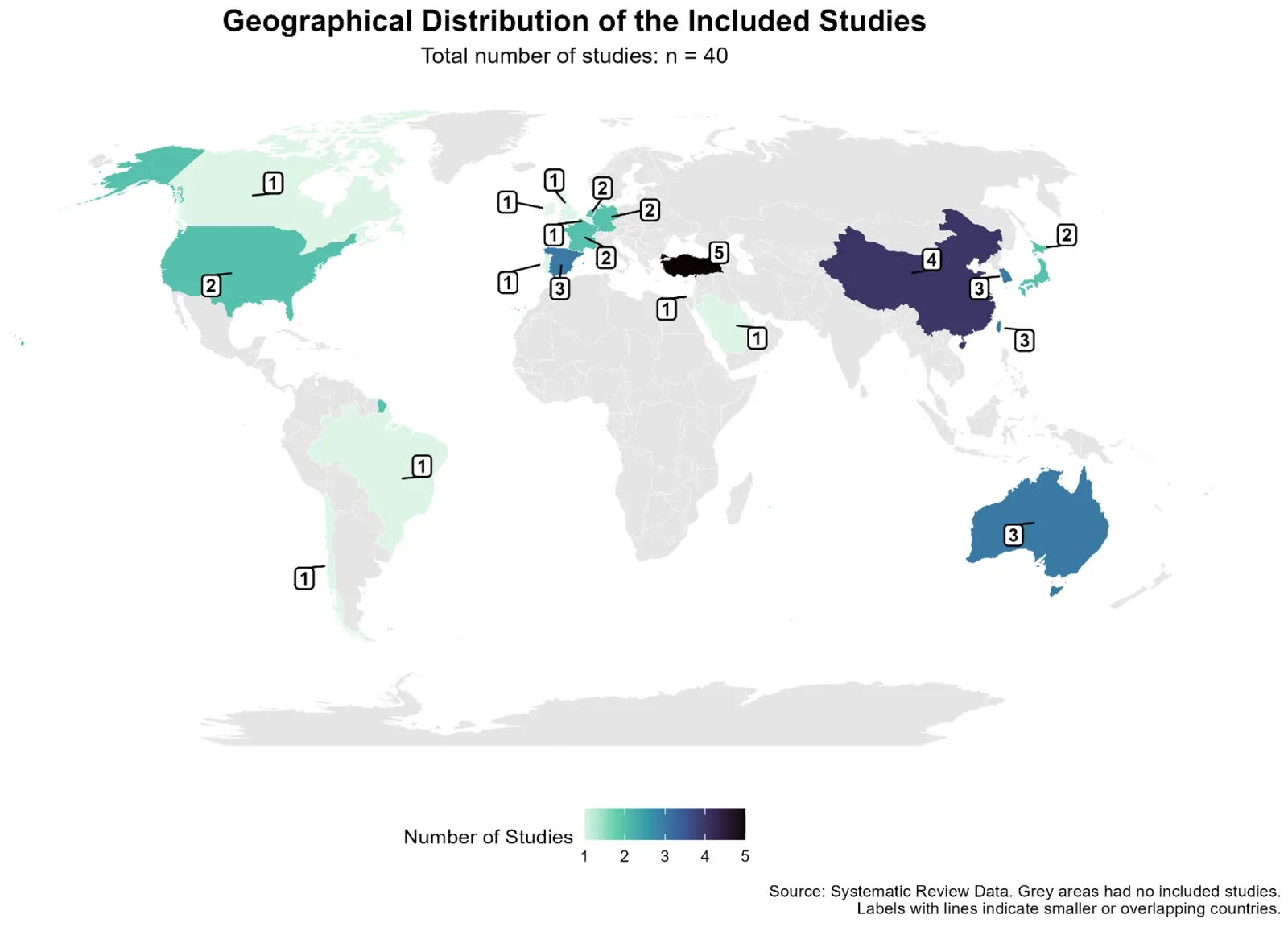
Geographical distribution of the included studies.

All 40 studies were conducted in the following countries: Turkey (n = 5), China (n = 4), Taiwan (n = 3), Spain (n = 3), Australia (n = 3), South Korea (n = 3), Germany (n = 2), France (n = 2), the Netherlands (n = 2), the USA (n = 2), and Japan (n = 2), with additional single-study contributions from Belgium, Brazil, Canada, Chile, Ireland, Israel, Portugal, Saudi Arabia and the United Kingdom. The included studies were geographically diverse, with a notable concentration from Asia (n = 19) and Europe (n = 13). America (n = 5) and Oceania (n = 3) were also represented. Figure 2 shows the geographical distribution of the included studies by country.

The primary characteristics of the included studies—such as study design, setting, participant demographics, outcome measures, and main findings—are summarized in Table 2. Various study designs were used, with cross-sectional studies (n = 22) being the most numerous. Longitudinal/cohort studies (n = 17) were also frequently observed, enabling analysis of vulnerability trajectories and predictors of change over time [54,56,58,60,62,69]. Another [31] was a quasi-experimental observational study with a multidomain intervention (n = 1).

Most of the studies selected for this review were set in community environments (n = 34; 85.0%), drawing samples from independent community-dwelling older adults, home-based services, or primary healthcare centers. A smaller proportion of studies (n = 4; 10.0%) were conducted in hospital or specialized outpatient clinic settings. The remaining 2 studies (5.0%) investigated frailty within long-term care settings, including residential aged care facilities and nursing homes.

Study cohorts ranged from small, focused groups (e.g., 71 to 74 participants) [35,46] to massive population-based datasets, such as those reported by Tsay et al. [59] (n = 16,309), Lee et al. [61] (n = 9171), and Tang et al. [48] (n = 9006).

The 40 included primary studies represent a cumulative total of 77,229 participant observations. This figure represents the aggregate sample sizes across published reports rather than unique individuals, as several investigations conducted distinct secondary analyses within shared national cohorts. Specifically, two studies derived from the Chinese Longitudinal Healthy Longevity Survey (CLHLS): Dong et al. [70] conducted a prospective longitudinal cohort study evaluating 4878 older adults newly enrolled in 2008 and followed across four survey waves (median follow-up: 65 months) to model incident frailty; whereas Tang et al. [48] performed a cross-sectional risk-modeling analysis of 9006 participants from the 2017–2018 wave using multivariable logistic regression. Similarly, two publications originated from the Toledo Study on Healthy Aging (TSHA) in Spain: Rodríguez-Laso et al. [58] examining multistate transitions across 1748 participants and Rodríguez-Laso et al. [64] focusing specifically on robustness-to-prefrailty transitions in a subset of 949 participants. In our synthesis, multiple publications originating from a single parent cohort were treated as complementary methodological evidence streams rather than independent population replications.

Regarding recruitment settings, studies were categorized into four distinct environments: (i) population-based community-dwelling independently sampled (n = 34); (ii) acute-care hospital inpatients or emergency department admissions (n = 4); and (iii) institutionalized residents in long-term care or nursing home facilities (n = 2).

**Figure 3:**
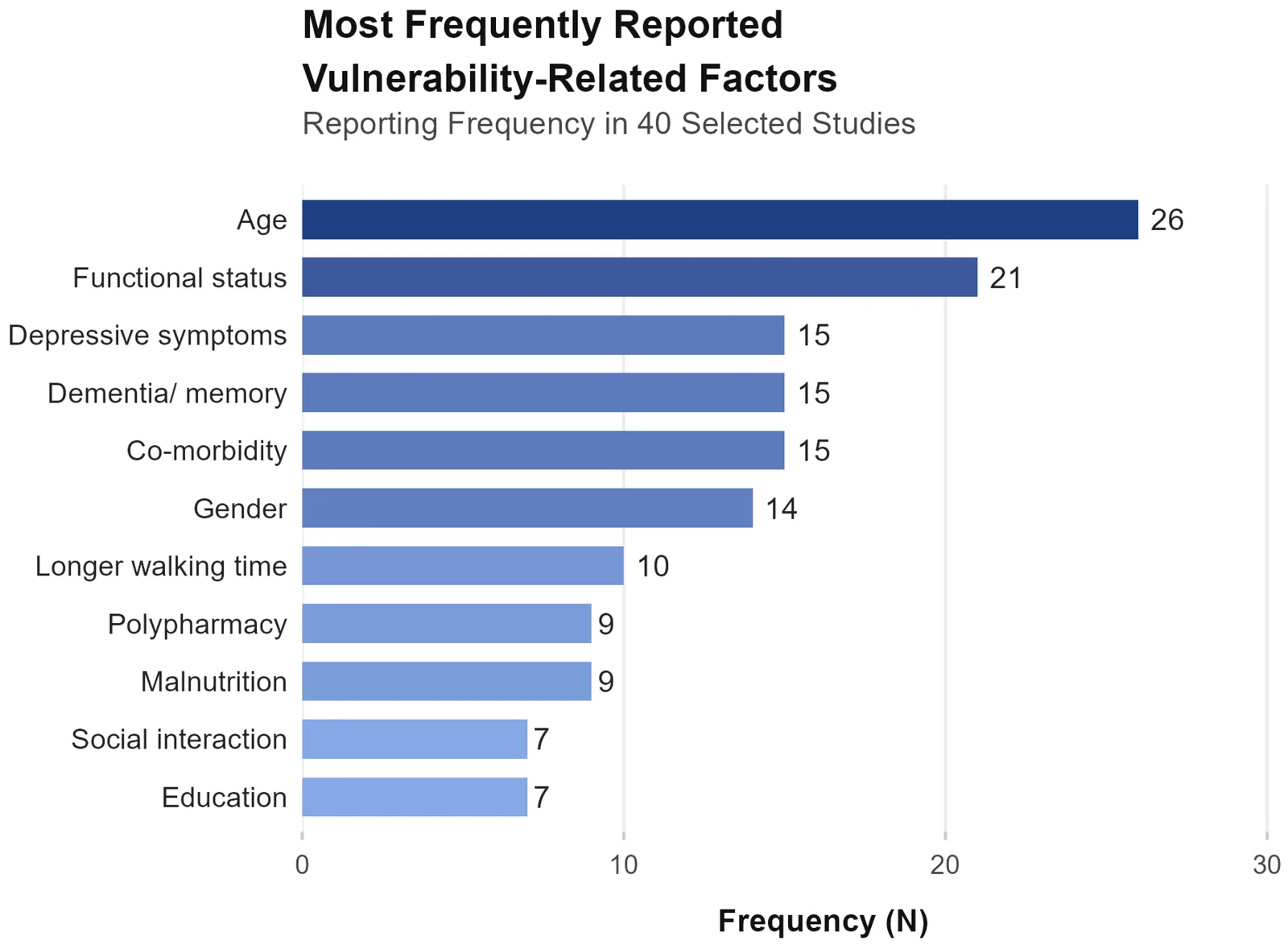
Most frequently reported vulnerability-related factors across included studies. Note: Frequencies indicate the number of primary investigations where candidate variables demonstrated independent, statistically significant associations (*p* < 0.05 or 95% CI excluding null values) in adjusted multivariable models, or were retained as independent predictive features in final multivariable/machine learning models (*N* = 40). Reporting frequency reflects observational prevalence across the published corpus rather than standardized effect magnitude, prognostic hierarchy, or causal precedence.

As illustrated in Figure 3, the analysis highlights the most frequently reported vulnerability-related factors across the included literature. Importantly, this ranking depicts reporting frequency across studies rather than the statistical magnitude, independence, or certainty of association. Advanced age was the most frequently reported factor (n = 26), followed by reduced functional status (n = 21). Psychological, cognitive, and clinical factors were also prominently represented, including depressive symptoms (n = 15), dementia or memory impairment (n = 15), and co-morbidity (n = 15). Gender-related differences were highlighted in 14 studies (consistently indicating a higher vulnerability prevalence among women). Other notable reported factors included objective mobility limitations, namely longer walking time (n = 10), polypharmacy (n = 9), malnutrition (n = 9), poor social interaction (n = 7), and lower educational attainment (n = 7).

Due to substantial heterogeneity across primary studies—including divergent definitions of vulnerability, differing covariates in multivariable models, and inconsistent reporting of metric effect sizes—pooled quantification of effect estimates (e.g., OR, HR) was not feasible. To address operational heterogeneity and distinguish concurrent correlates from prospective determinants, study-specific exposure metrics, outcome instruments, follow-up durations, multivariable-adjusted effect estimates (OR, HR, β) with 95% confidence intervals, and controlled covariates are systematically detailed across the three evidence streams in Table A5.

The studies analyzed in this review demonstrate substantial methodological diversity in the instruments used to operationalize vulnerability, reflecting the concept’s inherent complexity across varied care settings.

A comprehensive list of all other identified predictors is detailed in Table A4 (Appendix C).

## 4. Discussion

The findings of this systematic review synthesize the evidence on multidimensional factors associated with vulnerability and frailty in adults aged 65 and older across community, residential, and acute-care settings. To ensure rigorous interpretation in accordance with the methodological framework outlined in Section 2.6, extracted evidence was evaluated across three distinct epidemiological roles: (i) concurrent correlates identified in cross-sectional surveys; (ii) prospective predictors of frailty onset, progression, or state transitions in longitudinal cohorts; and (iii) baseline frailty as an exposure predicting downstream clinical adverse outcomes (institutionalization and mortality). Identified factors were organized across five overarching analytical domains (Table 3).

### 4.1. Biological Determinants

Chronological age was the most consistently evaluated demographic variable across the corpus (n = 26; Table A5). Prospective cohort investigations confirmed that advanced age significantly increases the risk of transitioning from robustness to prefrailty or frailty [56,58,66]. Rather than acting in isolation, age operates within a broader matrix of clinical decline, in which multimorbidity (n = 15) serves as a primary longitudinal predictor of functional decline and mortality [57,61] while showing high cross-sectional correlation with frailty severity in community and hospital samples [38,44,53].

Pharmacotherapeutic burden emerged as a prominent clinical determinant, though primary studies evaluated distinct operational exposures and epidemiological designs that must be clearly differentiated. General polypharmacy—predominantly operationalized as the concurrent use of five or more daily medications—demonstrated strong concurrent correlations with frailty severity across cross-sectional acute hospital and outpatient evaluations [34,44,47,50]. In prospective evaluations (Stream 2), baseline polypharmacy independently predicted adverse vulnerability trajectories: in the Longitudinal Aging Study Amsterdam (LASA), de Breij et al. [56] demonstrated that baseline polypharmacy (≥5 prescription drugs) conferred more than twice the odds of 3-year incident frailty (optimism-corrected aOR = 2.14, 95% CI: 1.53–4.19), retained in a final multivariable model adjusted for age, sex, number of chronic diseases, depressive symptoms, cognitive functioning, self-rated health, and pain (univariable OR = 4.76, 95% CI: 3.26–6.94). Similarly, continuous medication count independently hindered positive transitions from prefrailty back to robustness over long-term follow-up in the Toledo Study on Healthy Aging (TSHA) [64] (adjusted OR = 0.92 per additional drug, 95% CI: 0.86–0.99), while Lewis [55] identified high prescription counts (mean 8.24 drugs) and fall-risk-increasing drugs (≥2 FRIDs) as key markers of clinical instability in a community virtual ward cohort. However, interpreting numerical polypharmacy requires substantial caution regarding confounding by indication. Older adults are prescribed multiple medications to manage multimorbidity and advanced disease severity; although several included investigations adjusted for comorbidity indices (e.g., Charlson Comorbidity Index), residual confounding by underlying clinical severity and functional vulnerability cannot be excluded. Crucially, observational associations between high medication counts and frailty status cannot be construed as empirical evidence that deprescribing or reducing medication counts will reverse established frailty.

In contrast to simply counting medications, qualitative pharmacological properties provide a more specific prognostic perspective. In a 9-year follow-up cohort study of 1549 community-dwelling older adults, Tanaka et al. [69] assessed the burden of anticholinergic drugs using the validated Japanese Risk Scale. Older adults with a high baseline anticholinergic burden (score ≥ 3) exhibited a significantly elevated risk of incident physical frailty [adjusted HR = 2.45, 95% CI: 1.52–3.94] and sarcopenia [adjusted HR = 2.01, 95% CI: 1.20–3.35]. While this prospective finding confirms that anticholinergic burden operates as an independent observational marker of clinical decline, interventional randomized trials remain necessary to establish whether targeted pharmacological optimization can prevent or mitigate vulnerability trajectories.

### 4.2. Psychological Determinants

Depressive symptoms (n = 15) and cognitive decline (n = 15) were prominent psychological factors associated with vulnerability across the included corpus. In longitudinal cohort investigations, baseline depressive symptoms consistently emerged as independent prospective predictors of worsening frailty states, transitions from robustness to prefrailty, and adverse outcomes such as institutionalization and mortality [56,58,61,63,66,68]. Concurrently, cross-sectional surveys and quasi-experimental studies demonstrated high co-occurrence rates between depressive symptoms and physical frailty phenotypes [31,33,38,42,43,45,47,49,52,53]. Several investigations noted that depressive affect was linked to subsequent functional limitations, while worsening physical exhaustion reciprocally contributed to mood disturbances, suggesting a potential bidirectional relationship between affective state and clinical decline [33,38,49]. Cognitive impairment, ranging from subjective memory complaints to diagnosed dementia, was prospectively associated with adverse clinical trajectories in cohort studies, including accelerated functional dependency, residential admission, and mortality [54,55,56,58,60,61,66]. In cross-sectional evaluations, cognitive deficits showed marked concurrent associations with physical frailty markers and deficit accumulation [32,33,34,45,52]. Specifically, two studies explicitly operationalized the construct of ‘cognitive frailty’—defined as the simultaneous manifestation of physical frailty and cognitive impairment without overt dementia. In cross-sectional analysis, cognitive frailty was highly prevalent among older adults with lower educational attainment, malnutrition, and depressive symptoms [52]. Prospectively, longitudinal cohort evidence demonstrated that baseline factors such as advancing age, multi-morbidity, and lack of regular exercise significantly predict the clinical progression of cognitive frailty over time [68].

Poor self-rated health (n = 5) served as an integrative indicator of global vulnerability. In longitudinal cohorts, negative health perceptions independently predicted functional decline and frailty transitions [56,60], while cross-sectional investigations confirmed a linear relationship with physical frailty tiers across community and primary care samples [33,37,48]. Finally, sleep disturbances (n = 2), characterized by poor sleep quality or irregular patterns, were associated with higher frailty odds in community-based cohorts [48,70], reflecting disruptions in neuroendocrine and circadian homeostasis that commonly accompany clinical decline.

### 4.3. Socioeconomic/Relational Determinants

The synthesized literature identified consistent patterns across sociodemographic factors. Female gender was repeatedly identified as an associated factor with a higher prevalence of clinical frailty across cross-sectional investigations [34,35,38,41,47,50,52]. In contrast, longitudinal cohort studies observed that male older adults, despite demonstrating lower baseline frailty prevalence, experienced faster clinical deterioration and higher mortality rates [60,61] or demonstrated distinct transition trajectories between robust and frail states [56,65,68]. This divergence is consistent with the recognized male-female health-survival paradox in gerontological epidemiology. Lower educational attainment (n = 7) and socioeconomic hardship (n = 4) were recurring social determinants associated with vulnerable states. Dong et al. [70] conducted a prospective cohort study which found that lower levels of education and material deprivation were associated with an increased risk of frailty progression, transitions to functional disability and higher mortality. Similarly, population-based cross-sectional studies documented that limited formal education and neighborhood-level material deprivation were strongly associated with higher odds of both physical and cognitive frailty [32,41,48,50,52,53]. Furthermore, racial and ethnic minority backgrounds were identified in three studies as factors associated with cumulative psychosocial adversity and elevated frailty risk [41,48,70]. Relational determinants, including reduced social interaction (n = 7), living alone (n = 3), unmarried or widowed status (n = 3), and infrequent visitation (n = 2), were consistently associated with vulnerable phenotypes. In longitudinal investigations, diminished social participation and restricted community support networks independently predicted adverse frailty transitions and institutionalization [59,63,65]. Cross-sectional studies link “living without a partner”, “loneliness”, and “relational distance” to physical vulnerability and a reduced quality of life [33,34,42,43,48]. These associations, described as “social frailty,” may have a dimension that goes beyond the biological and may be important for overall vulnerability.

### 4.4. Behavioral/Lifestyle Determinants

Modifiable lifestyle factors display consistent associations with vulnerability and frailty trajectories across the reviewed literature. Sedentary behavior and insufficient physical activity were identified as significant risk factors for functional decline and frailty progression across prospective cohorts [58,59,67,68,69,70] while being associated with phenotypic frailty in cross-sectional and quasi-experimental evaluations [31,48,53]. Conversely, engagement in regular exercise and health-promoting behaviors was associated with preserved functional status, psychological resilience, and a higher probability of maintaining or regaining robustness in longitudinal and intervention studies [65,67,68]. Cigarette smoking was identified as an independent longitudinal predictor of adverse frailty transitions and accelerated clinical deterioration in prospective cohort designs [58,70]. In parallel, alcohol consumption patterns were evaluated across two investigations [48,64]: prospective cohort evidence from the TSHA study demonstrated that non-drinking was associated with frailty state transitions [64], while cross-sectional multivariable modeling in the CLHLS identified alcohol intake patterns within the broader lifestyle risk profile [48]. Furthermore, lower health literacy was identified by Yoshizawa et al. [63] in a prospective cohort as an independent predictor of frailty progression over 4 years. In that study, limited health literacy compromised older adults’ capacity to comprehend health information and navigate health resources, highlighting a key target for preventive educational and nursing interventions in primary care.

### 4.5. Self-Care Profile and Functional Determinants

Within the self-care profile, functional limitations in activities of daily living (ADLs and IADLs) represented the most prevalent functional indicators across the included literature. In prospective cohort studies, baseline limitations in instrumental ADLs (e.g., medication management, meal preparation, financial administration) and basic ADLs were identified as independent longitudinal predictors of frailty progression, institutionalization, and all-cause mortality [55,59,61,65,67,69,70]. Cross-sectional surveys and clinical evaluations further confirmed strong concurrent associations between functional dependence and frailty across community and acute-care populations. Notably, Costenoble et al. [65] demonstrated that restrictions in advanced ADLs (a-ADLs) and instrumental tasks precede basic ADL dependence, functioning as early indicators of transitions from robustness to prefrailty and frailty. Objective measures of physical performance and neuromuscular capacity—including decreased gait speed, longer walking time, and reduced or asymmetric handgrip strength—served as sensitive clinical markers of sarcopenia and biological decline. In prospective evaluations, impaired walking time and reduced muscle strength prospectively predicted frailty worsening and adverse health transitions [58,69]. In parallel, cross-sectional and sensor-based biomechanical studies highlighted that handgrip strength weakness and gait asymmetry reliably distinguish frail from non-frail older adults [35,39,46,47,50,51,53]. Nutritional compromise (n = 9), identified via validated screening instruments (e.g., MNA, MUST), was consistently associated with frailty onset and progression [31,32,34,36,47,49,50,52,62]. In residential aged care, Thompson et al. [62] identified malnutrition and risk of malnutrition as prospective predictors of 12-month frailty worsening, while cross-sectional studies documented significant associations between poor nutritional status and physical exhaustion [34,36,49,52]. This vulnerability was compounded by age-related swallowing difficulties and presbyphagia (n = 3), which were correlated with depressive symptoms, inadequate caloric intake, and exacerbated physical frailty [36,49,63]. Nutritional and functional decline was frequently mirrored by objective laboratory biomarkers: low serum albumin levels were prospectively predictive of mortality and frailty progression [57] and cross-sectionally linked to frailty severity [39]. Furthermore, hematological markers—including lower hemoglobin concentrations, elevated C-reactive protein (CRP), and a reduced hemoglobin-to-red blood cell distribution width ratio (HRR)—correlated with physical exhaustion, chronic systemic inflammation, and muscle weakness [32,40]. Finally, psychophysical concerns heavily intersected with functional capacity. A documented history of falls and fear of falling (n = 5) were correlated with voluntary activity restriction and deconditioning, contributing directly to functional dependency [33,52,53,55,63]. Chronic pain (n = 1), identified as an independent predictor of frailty in the LASA cohort [56], restricted mobility and compromised self-care routines. Pressure injury risk was similarly identified as a clinical marker reflecting severe immobility, malnutrition, and advanced clinical dependence [55].

### 4.6. Conceptual Heterogeneity and Methodological Synthesis

A critical finding emerging from this systematic review is the marked conceptual and operational heterogeneity across primary studies. Although our review conceptualizes vulnerability as an overarching biopsychosocial state, primary investigations predominantly operationalized risk through specific frailty instruments:

The Physical Phenotype: Studies utilizing Fried’s criteria focused exclusively on biological and physiological dimensions (exhaustion, unintentional weight loss, low grip strength, slow walking speed, low physical activity), assessing vulnerability through the lens of physiological exhaustion and sarcopenia [31,32,37,39,41,46,53,58,60,65,66].

Deficit Accumulation (Frailty Index): Investigations adopting the Frailty Index (FI) conceptualized vulnerability as a mathematical ratio of accumulated clinical, functional, and psychological deficits across multiple physiological systems [45,48,59,62,70].

Rapid Clinical and Inpatient Scales: Studies set in acute-care, emergency, or hospital wards prioritized rapid screening instruments, such as the Clinical Frailty Scale (CFS) [38,44,47,50,55] and the Edmonton Frail Scale (EFS) [34,49,57], which blend functional mobility with clinical judgement.

Multidimensional and Domain-Specific Tools: A smaller subset of studies deployed instruments that incorporate social and psychological indicators, such as the Tilburg Frailty Indicator (TFI) [42,67] and the Kihon Checklist (KCL) [63], or isolated sub-constructs such as “cognitive frailty” [52,68] and “social frailty” [42,43].

This operational divergence illustrates that while primary literature frequently employs “frailty” as an operational surrogate for vulnerability, frailty scales primarily measure biological reserve and physical performance. True multidimensional vulnerability necessitates integrating social support, cognitive capacity, environmental conditions, and self-care profiles alongside traditional physical phenotypes.

Finally, these synthesized determinants must be interpreted in light of the underlying methodological designs. As detailed in the results, more than half of the evidence base relies on analytical cross-sectional studies; consequently, these findings reflect concurrent associations and shared vulnerability profiles rather than causal pathways or temporal precedence. True prospective prognostic capability is restricted to the candidate determinants evaluated and validated within the 17 longitudinal cohort investigations.

A critical methodological caveat identified in this synthesis concerns predictor–outcome construct overlap (incorporation bias). Several frequently reported physical performance factors—most notably gait speed, handgrip strength, and physical exhaustion—serve simultaneously as candidate predictor variables and as core diagnostic criteria in phenotypic frailty instruments (such as the Fried phenotype). Consequently, statistical associations between these neuromuscular parameters and frailty status in cross-sectional and unadjusted models partially reflect mathematical and conceptual circularity rather than independent etiological prediction. In contrast, extrinsic multidimensional determinants—including social detachment, educational deprivation, depressive mood, and pharmacotherapeutic burden—operate independently of physical diagnostic definitions. Rather than asserting superior prognostic performance, these multidimensional determinants are less susceptible to predictor–outcome incorporation bias and provide complementary prognostic information alongside established physical phenotypes to support comprehensive clinical risk evaluation.

### 4.7. Limitations

Although this systematic review provides a comprehensive evidence-based synthesis of multidimensional reported risk factors in older adults, several methodological and contextual limitations must be acknowledged.

First, from a design perspective, more than half of the included evidence base (n = 22) relies on analytical cross-sectional designs, precluding causal inference and the determination of temporal precedence between candidate determinants and vulnerability states. While 17 longitudinal cohort studies were analyzed, their follow-up horizons varied substantially—ranging from 12 months in specific residential cohorts [62,65] to 9 years in population registries [69]—which may differentially capture acute transitional fluctuations versus insidious, long-term biological decline.

Second, the cumulative sample of 77,229 participant observations reflects aggregate study reports rather than entirely unique individuals. Two included investigations performed distinct, complementary analyses on shared national databases (specifically, the CLHLS in China [48,70] and the TSHA in Spain [58,64]. While their analytical designs and outcome frameworks differed, this shared provenance means that population-level evidence is partly concentrated in specific cohorts. Furthermore, many primary studies excluded older adults with severe dementia, total functional dependency, or acute critical illness, potentially underestimating vulnerability prevalence and risk burdens in the most vulnerable strata.

Third, marked conceptual and operational heterogeneity across frailty instruments (ranging from physical phenotypes and deficit accumulation indices to rapid emergency screening tools) precluded quantitative meta-analysis. Consequently, our synthesis matrix (Table 3 and Table A5) reflects the reporting frequency of candidate variables across published studies rather than a standardized hierarchy of statistical effect sizes or independent prognostic weights. Relatedly, while our review conceptualized vulnerability as an overarching biopsychosocial state, primary research predominantly operationalized risk through physical frailty phenotypes. Although physical frailty provides an established, clinically translatable proxy for adverse geriatric outcomes [8,23,25], it captures only a portion of the broader multidimensional vulnerability construct.

Fourth, information and reporting biases must be considered. The reliance on self-reported questionnaires for behavioral, lifestyle, nutritional, and relational variables across multiple investigations introduces potential recall and social desirability biases, whereas objective laboratory biomarkers were evaluated in only a small subset of hospital-based studies. Additionally, restricting our systematic search to peer-reviewed articles published between 2020 and 2025 in four languages (English, French, Portuguese, and Spanish) may have omitted relevant earlier benchmark investigations or literature published in other linguistic regions.

Finally, while we acknowledge the critical issue of predictor–outcome overlap (incorporation bias)—particularly between physical performance predictors and physical frailty diagnostic criteria—we were unable to assess this overlap separately for each specific multidimensional instrument and measurement time point. Because we could not statistically isolate and compare the prognostic robustness of physical versus nonphysical determinants after accounting for overlapping variables, this remains an unresolved limitation of the current synthesized evidence.

### 4.8. Future Research

Future research should prioritize prospective studies integrating objective digital phenotyping (such as wearable sensors for gait kinematics and mobility) alongside inflammatory and nutritional biomarkers (e.g., CRP, albumin) to minimize self-report bias and clarify pathophysiological trajectories. Furthermore, investigators should utilize the multidimensional candidate variables synthesized in this review to develop and externally validate machine learning algorithms, rigorously assessing model calibration, discrimination, and clinical utility while avoiding predictor–outcome incorporation bias. Finally, randomized interventional trials are needed to establish whether targeted deprescribing and the systematic reduction in anticholinergic burden can actively mitigate vulnerability trajectories across clinical and community care settings.

## 5. Conclusions

This systematic review synthesized the multifaceted determinants associated with vulnerability and frailty in adults aged 65 and older across community, residential, and acute-care settings. A central insight emerging from this evidence base is that primary research rarely operationalizes vulnerability as a uniform, single outcome. Instead, investigations evaluate risk through distinct frailty paradigms—ranging from physical phenotype models and cumulative deficit indices to multidimensional tools and domain-specific constructs, such as cognitive or social frailty.

Advanced age was the most consistently documented non-modifiable factor. Across biological and clinical dimensions, multimorbidity and numerical polypharmacy correlated with greater frailty severity and prospectively predicted adverse trajectories, including institutionalization and mortality, though these associations remain subject to confounding by indication. Furthermore, longitudinal cohort evidence highlighted qualitative pharmacological risk, demonstrating that high baseline anticholinergic burden independently predicts incident physical frailty over time.

Within the psychological domain, depressive symptoms and cognitive decline were repeatedly linked to vulnerability, independently predicting accelerated functional loss and mortality. When cognitive impairment co-occurs with physical frailty—defined operationally as cognitive frailty—it is prospectively associated with an elevated risk of progressive clinical deterioration. Poorer self-rated health and persistent sleep disturbances further delineate a psychophysical profile that parallels vulnerable states.

Socioeconomic and relational determinants also proved pivotal. Lower educational attainment, financial hardship, and racial or ethnic disparities were associated with heightened vulnerability, reflecting cumulative life-course inequities and limited health literacy. Concurrently, relational detachment—operationalized through living alone, widowhood, and restricted social interaction—consistently correlated with adverse physical outcomes and compromised quality of life, underscoring social frailty as an indispensable component of comprehensive geriatric assessment.

Regarding functional and self-care profiles, reduced gait speed, handgrip weakness, step asymmetry, and nutritional deficits (malnutrition, low serum albumin) were among the most prominent indicators. Rather than operating as isolated prospective causes, objective physical performance parameters largely function as sensitive indicators of depleted physiological reserve and, in physical frailty phenotypes, partially represent operational diagnostic criteria (incorporation bias). Notably, longitudinal findings indicate that functional decline typically manifests in advanced and instrumental activities of daily living before progressing to basic self-care dependence.

Critically, this systematic review does not deliver an empirically validated prediction algorithm or an established clinical risk-stratification instrument. Instead, it provides an evidence-based synthesis and categorization of candidate variables across biological, functional, psychological, behavioral, and relational domains. Future research must prioritize large, prospective longitudinal cohorts and advanced machine learning techniques to externally validate these candidate features, evaluate their statistical independence, and determine their calibration and clinical net benefit. Such validation will be essential for translating these observational determinants into precise, person-centered risk screening in clinical practice.

## Figures and Tables

**Table 1:** Summary of methodological quality and risk of bias across included studies.

Study	JBI Appraisal Tool	Final Score	Quality Level
Huang et al. (2023) [31]	Quasi-Experimental	6/9 (67%)	Moderate
Steinmeyer et al. (2020) [32]	Cross-sectional	8/8 (100%)	High
Venturini et al. (2021) [33]	Cross-sectional	6/8 (75%)	Moderate
Sharma et al. (2021) [34]	Cross-sectional	8/8 (100%)	High
Jung et al. (2021) [35]	Cross-sectional	8/8 (100%)	High
Sella-Weiss et al. (2021) [36]	Cross-sectional	8/8 (100%)	High
Tavares et al. (2022) [37]	Cross-sectional	8/8 (100%)	High
Mangin et al. (2023) [38]	Cross-sectional	6/8 (75%)	Moderate
Yan et al. (2023) [39]	Cross-sectional	8/8 (100%)	High
Zhu et al. (2023) [40]	Cross-sectional	8/8 (100%)	High
Shakya et al. (2023) [41]	Cross-sectional	8/8 (100%)	High
Cömertoğlu et al. (2023) [42]	Cross-sectional	7/8 (88%)	High
Henry et al. (2023) [43]	Cross-sectional	8/8 (100%)	High
Alamri & Mealif (2024) [44]	Cross-sectional	8/8 (100%)	High
Johnson et al. (2024) [45]	Cross-sectional	8/8 (100%)	High
Sepúlveda et al. (2024) [46]	Cross-sectional	8/8 (100%)	High
Güner et al. (2024) [47]	Cross-sectional	8/8 (100%)	High
Tang et al. (2024) [48]	Cross-sectional	8/8 (100%)	High
Savci et al. (2025) [49]	Cross-sectional	8/8 (100%)	High
Ceylan et al. (2025) [50]	Cross-sectional	8/8 (100%)	High
Ortiz-Navarro et al. (2025) [51]	Cross-sectional	8/8 (100%)	High
Park et al. (2025) [52]	Cross-sectional	8/8 (100%)	High
Tzeng et al. (2025) [53]	Cross-sectional	8/8 (100%)	High
Hajek et al. (2020) [54]	Cohort	8/11 (73%)	Moderate
Lewis (2020) [55]	Cohort	7/11 (64%)	Moderate
Breij et al. (2020) [56]	Cohort	8/11 (73%)	Moderate
Varan et al. (2021) [57]	Cohort	10/11 (91%)	High
Rodríguez-Laso et al. (2021) [58]	Cohort	8/11 (73%)	Moderate
Tsay et al. (2021) [59]	Cohort	9/11 (82%)	High
Mielke et al. (2022) [60]	Cohort	9/11 (82%)	High
Lee et al. (2022) [61]	Cohort	11/11 (100%)	High
Thompson et al. (2022) [62]	Cohort	9/11 (82%)	High
Yoshizawa (2022) [63]	Cohort	9/11 (82%)	High
Rodríguez-Laso et al. (2023) [64]	Cohort	9/11 (82%)	High
Costenoble et al. (2023) [65]	Cohort	9/11 (82%)	High
Tchalla et al. (2023) [66]	Cohort	9/11 (82%)	High
Van der Ploeg et al. (2024) [67]	Cohort	8/11 (73%)	Moderate
Suprawesta et al. (2024) [68]	Cohort	9/11 (82%)	High
Tanaka et al. (2025) [69]	Cohort	10/11 (91%)	High
Dong et al. (2025) [70]	Cohort	10/11 (91%)	High

**Table 2:** Characteristics of included studies.

Authors (Year)	Country	Study Design	Study Aim	Setting	Participants and Sample	Outcome Measures	Main Findings
**Hajek et al. (2020) [54]**	Germany	Cohort	Predictors of frailty among individuals aged 85+	Community (Primary care)	486 individuals aged ≥85 (88.7 ± 2.9 years)	Clinical Frailty Scale (CFS)	Age ↑, dementia ↑ (significant), chronic conditions ↑ (marginally significant)
**Lewis (2020) [55]**	Ireland	Cohort	Health trajectories and patient-specific factors influence the risk of adverse outcomes (hospitalization, institutionalization, death).	Community Virtual Ward	88 older adults (82.8 ± 6.4 years).	Health status indicators, disability, and adverse clinical outcomes	Cognition and function were key predictors; delirium and multiple care episodes predicted institutionalization
**Steinmeyer et al. (2020) [32]**	France	Cross-sectional study	To evaluate whether haemoglobin concentration is an independent predictor of frailty, and to investigate causal pathways	Hospital	1829 individuals aged ≥65 (82.4± 6.5 years)	Fried’s frailty phenotype	↓ haemoglobin concentration, inflammation (CRP), cognitive impairment, malnutrition, and ↓ education level
**Breij et al. (2020) [56]**	Netherlands	Cohort study	To identify predictors of frailty and vitality in older adults aged 75 years and over, the Longitudinal Ageing Study Amsterdam (LASA)	Community-dwelling	569 individuals aged ≥75 (79.4 ± 5.4 years)	Frailty and vitality (LASA Frailty Index)	Age, depressive symptoms, ↑ chronic diseases, poor self-rated health, cognitive functioning, polypharmacy, and pain
**Venturini et al. (2021)** **[33]**	Brazil	Cross-sectional study	To create a multidimensional model to evaluate frailty in older Brazilian adults and to compare the dimensions of the model created among the categories of the physical frailty phenotype (FIBRA-BR).	Community-dwelling	3569 participants (73.7 ± 6.6 years).	Frailty (Physical frailty phenotype)	Poor self-rated health, urinary incontinence, not visiting/receiving visitors, depressive symptoms, falls, feelings of sadness, and cognitive impairment
**Varan et al. (2021) [57]**	Turkey	Cohort study (prospective)	To compare the predictive value of all comprehensive geriatric assessments	Community-dwelling	967 patients aged ≥65 (median 73 years)	Edmonton Frail Scale and Fried Frailty Index/Long-term mortality/	Serum albumin level, ↓ functional status (IADL) score, and walking time were identified as the best predictors
**Sharma et al. (2021) [34]**	Australia	Cross-sectional	To determine whether the Malnutrition Universal Screening Tool (MUST) can predict frailty in older hospitalized patients	Hospital	243 patients ≥ 65 (83.9 ± 6.5 years)	Frailty (Edmonton Frail Scale)	limitations in (ADL), sex, social participation, age, cognitive impairment, ↓ independence in mobility, ↑ comorbidity and polypharmacy
**Jung et al. (2021) [35]**	South Korea	Cross-sectional study	To assess frailty using gait parameters and LSTM networks	Community setting with wearable sensors	74 elderly adults aged ≥65 (76 ± 4 years)	Frailty classification (FRAIL scale & Machine Learning)	Gait variability, asymmetry, irregularity in step/stride/double-limb support times; demographic factors (age, sex)
**Sella-Weiss et al. (2021) [36]**	Israel	Cross-sectional study	To explore associations between swallowing function, dysphagia, malnutrition, depression and frailty	Community-dwelling	180 individuals aged ≥65 (75.9 ± 7.9 years)	Frailty (FRAIL Questionnaire)	Age ↑, suspected dysphagia ↑, depression ↑, poor nutrition ↑; worse swallowing performance
**Rodríguez-Laso et al. (2021) [58]**	Spain	Cohort study (prospective)	To examine the frequency and predictors of transitions between frailty states in community-dwelling older adults	Community-dwelling (Toledo Study on Healthy Ageing—TSHA)	1748 individuals aged ≥65 (75.0 ± 5.9 years)	Fried phenotype	age, smoking, depressive symptoms, hearing/cognitive impairment, and ↓ physical activity
**Tsay et al. (2021) [59]**	USA	Cohort study	To identify predictors of change in frailty status.	Community-based Home and Community-Based Services (HCBS)	16,309 older adults ≥ 65 (80 years)	Clinically meaningful changes in Frailty Index (FI) score	age ↑, ↓ ADL and physical activity, service use (in-home care, adult day), living alone, social interaction
**Tavares et al. (2022) [37]**	Portugal	Cross-sectional study	To identify the prevalence and predictors of frailty (Primary Health Care)	Community-dwelling	136 older adults aged ≥65 (74.0 ± 6.2 years)	Frailty (Fried frailty phenotype)	Age ↑, self-rated health, physical capacity
**Mielke et al. (2022) [60]**	Germany	Cohort study	To analyze gender differences in frailty transition patterns and develop prediction models for improvement/worsening	Community-dwelling older	1158 adults aged ≥70 (84.4 ± 5.6 years)	Frailty (Fried frailty phenotype)	Gender, age, cognitive impairment, education, self-rated health, diabetes, stroke, congestive heart failure (CHF), chronic kidney disease (CKD), osteoarthritis, hospitalization, living alone
**Lee et al. (2022) [61]**	South Korea	Cohort study	To investigate how multiple frailty domains and their combinations predict institutionalization and mortality (Living Profiles of Older People Survey—LPOPS)	Community-based sample	9171 adults aged ≥65 (73.1 ± 6.8 years)	Multiple dimensions (physical, cognitive, psychological, and social) profiles; Institutionalization and all-cause mortality	age, gender, education, income, marital status, comorbidities, health behaviors; multiple dimensions predict institutionalization/mortality
**Thompson et al. (2022) [62]**	Australia	Cohort study	To estimate the minimally important difference (MID) in frailty, then describe frailty change over 12 months, and identify factors associated with worsening frailty.	Long-term care/Residential Aged Care Services (RACS)	548 residents aged ≥65 (87.7 ± 7.2 years).	Frailty change (Frailty Index)	age, malnutrition, risk of malnutrition, and diabetes
**Yoshizawa (2022) [63]**	Japan	Cohort study	To examine whether health literacy (HL) is a predictor of frailty progression among community-dwelling older adults.	Community (12 local centers and health institutions).	621 participants (71.6 ± 4.6 years).	Frailty progression (Kihon Checklist)	Age ↑, ↓ HL, poor mental health, lack of social support, negative views toward mutual aid of social capital
**Rodríguez-Laso et al. (2023) [64]**	Spain	Cohort study	To explore predictors of sustained transitions between robustness and prefrailty in both directions among older adults	Community-dwelling (Toledo Study on Healthy Ageing—TSHA)	949 individuals aged ≥65 (72.3 ± 7.1 years)	Fried phenotype	age, ↑ medications, not declaring alcohol consumption, and ↓ grip strength
**Mangin et al. (2023) [38]**	Canada	Cross-sectional study	To explore the association between frailty, chronic conditions, and socioeconomic status (SES) among older adults attending primary care, McMaster University Sentinel and Information Collaboration (MUSIC).	Community Primary care	2043 adults aged ≥65 (76.0 ± 7.8 years)	Frailty (Clinical Frailty Scale)	age, female sex, multimorbidity, ↓ neighborhood income, and ↑ neighborhood material deprivation
**Yan et al. (2023) [39]**	China	Cross-sectional study	To explore the relationship between frailty syndrome and blood pressure variability in the elderly.	Hospital (Neurology department)	150 patients aged ≥65 (69.79 ± 3.23 years)	Senile debilitating syndrome/Frailty (Fried scale)	Risk factor: Age. Protective factors: Serum albumin (ALB) and high-density lipoprotein (HDL-C)
**Huang et al. (2023) [31]**	Taiwan	Quasi-experimental study	To investigate the associations between physical activity, frailty, and the effects of multidomain intervention.	Community	106 older adults aged 65–96 (77.47 ± 7.19 years)	Frailty (Fried’s frailty phenotype)	↓ physical activity (assessed via PASE scores), ↓ mobility, and more depressive symptoms
**Zhu et al. (2023) [40]**	China	Cross-sectional population-based study	To investigate the association between haemoglobin-to-red blood cell distribution width ratio (HRR) and frailty in older adults.	Community-dwelling	1296 older adults aged ≥65 (70.89 ± 4.85 years)	Frailty (Fried Frailty Phenotype)	The haemoglobin-to-red blood cell distribution width ratio (HRR)is a good predictor.
**Costenoble et al. (2023) [65]**	Belgium	Cohort study	To examine whether changes in basic, instrumental, and advanced activities of daily living (b-, i-, a-ADLs), social participation, and psychological resilience predict both a transition from robustness to prefrailty or frailty and vice versa	Community-dwelling	322 octogenarians (83.04 ± 2.78 years)	Worsened and improved frailty transitions (adapted Fried phenotype)	*Worse:* limitations (a-ADLs); *improved:* Female gender; ↑ social participation
**Shakya et al. (2023) [41]**	USA	Cross-sectional	To examine the mediating role of cumulative psychosocial stress on frailty disparities among older adults	Community-dwelling	7679 older adults aged ≥65	Frailty (Fried frailty phenotype)	Female gender, ethnicity, ↓ education, and ↑ cumulative psychosocial stress (e.g., loneliness, discrimination, financial strain)
**Tchalla et al. (2023) [66]**	France	Cohort study	To identify frailty trajectories and their baseline predictors and clinical outcomes, FREEDOM	Community-dwelling	497 individuals aged >75 or >65 with ≥2 comorbidities (82.7 ± 5.5 years)	Frailty trajectories (Fried’s criteria)	Age, cognitive deficit/dementia, depressive symptoms, and hypertension
**Cömertoğlu et al. (2023) [42]**	Turkey	Cross-sectional study	To examine the effect of social frailty on mental health and quality of life in older people.	Community-dwelling	136 participants over 65 years old (72.2 years median)	Social frailty (Tilburg Frailty Indicator); Quality of life	Being widowed, having anxiety symptoms, and increased physical limitations
**Henry et al. (2023) [43]**	Australia	Cross-sectional study	To provide initial insights into whether social cognitive difficulties in older age are associated with social frailty, and how social frailty is linked to psychological characteristics important for health and well-being.	Community-dwelling	90 participants aged between 65 and 91 (73.78 ± 5.92 years)	Social frailty (Social Frailty Scale)	Poorer (inappropriate) social behavior, ↓affective empathy, and depression
**Van der Ploeg et al. (2024) [67]**	Netherlands	Cohort study	To examine transitions in disability over nine years among Dutch community-dwelling older people aged ≥75 and determine associated predictors	Community-dwelling	479 participants at baseline, 69 with complete data across 9 years	Disability transitions, Tilburg Frailty Indicator	Lifestyle (‘not healthy’), diseases or chronic disorders (‘two or more’), age, income, marital status, education, life events
**Suprawesta et al. (2024) [68]**	Taiwan	Longitudinal cohort study	To identify baseline predictors associated with cognitive frailty (CF) improvement or progression in community-dwelling older adults	Community-dwelling	832 adults aged ≥65 (70.9 ± 5.1 years) (482 completed 2-year follow-up)	Cognitive frailty (CF) progression	Age, sex, Tinetti balance score, gait velocity, number of comorbidities, depression, regular exercise
**Alamri & Mealif (2024) [44]**	Saudi Arabia	Cross-sectional	To estimate frailty prevalence and identify its predictors among hospitalized older adults	Hospital	147 inpatients aged ≥65 years	Frailty status (Clinical Frailty Scale)	Higher comorbidity index, polypharmacy, heart failure, and shorter height.
**Johnson et al. (2024) [45]**	United Kingdom	Cross-sectional	To explore the subdimensions of frailty index using factor analysis and their relationship to quality of life, English Longitudinal Study of Ageing (ELSA)	Community-based	4971 participants ages ≥ 65 (74.9 ± 7 years)	Quality of life/Frailty Index subdimensions	mobility impairment & physical morbidity, difficulty in daily activities, mental health, disorientation
**Sepúlveda et al. (2024) [46]**	Chile	Cross-sectional study	To identify oxylipins as potential plasma biomarkers for frailty syndrome in ageing, especially in women	Community centers and health clinics	71 older adults (F = 50, M = 21), aged ≥65 (F = 73.6 ± 5.9, M = 76.2 ± 6.2 years)	Frailty (Fried Phenotype & FTS-5)	↑ plasma oxylipin levels—specifically thromboxane B2 (TxB2) and 7-Hydroxydocosahexaenoic acid (HDoHE) in women—correlated with poor grip strength and slow gait.
**Güner et al. (2024) [47]**	Turkey	Cross-sectional	To evaluate whether handgrip strength (HGS) asymmetry and weakness are independent predictors of frailty in older adults	Community-dwelling	512 older adults aged ≥65 (73.2 ± 6.1 years)	Frailty (Clinical Frailty Scale)	Handgrip strength (HGS) asymmetry and overall muscle weakness.
**Tang et al. (2024) [48]**	China	Cross-sectional	To identify frailty risk factors and construct a predictive nomogram for older adults in the Chinese Longitudinal Healthy Longevity Survey (CLHLS)	Community-dwelling,	9006 adults aged ≥65 (83.2 years)	Frailty risk (Frailty Index)	*Risk factors:* age, ↓ weight or obesity, ↓ self-assessed economic status, and poor sleep quality. *Protective factors:* education, having a spouse, basic living security, exercising, and social activities.
**Savci et al. (2025) [49]**	Turkey	Cross-sectional	To examine relationships between dysphagia risk, frailty, and depression in older adults in nursing homes	Long-term care (Nursing home)	205 older adults aged ≥65 (70.65 ± 6.36 years)	Frailty (Edmonton Frailty Scale)	Risk of dysphagia (swallowing difficulties) and advanced age
**Tanaka et al. (2025) [69]**	Japan	Longitudinal cohort study	To investigate whether anticholinergic drug burden predicts frailty and sarcopenia in community-dwelling older adults, 9-year follow-up	Community-dwelling	1549 adults aged ≥65 (72.5 ± 5.5 years)	Frailty and sarcopenia	Anticholinergic drug burden
**Ceylan et al. (2025) [50]**	Turkey	Cross-sectional study	To investigate whether handgrip strength (HGS) can alone detect individuals living with frailty (LWF) using the Clinical Frailty Scale (CFS)	Community-dwelling	742 patients aged ≥65; 49.3% LWF (median 72 years)	Frailty (Clinical Frailty Scale)	Older age, female sex, and lower handgrip strength.
**Ortiz-Navarro et al. (2025) [51]**	Spain	Cross-sectional study	To identify body composition predictors of frailty using DXA, BIA, and US and assess their feasibility in primary care with a mobile app (PowerFrail App)	Community-dwelling	94 older adults aged 70–80 classified as frail, prefrail, or robust (76.46 ± 2.92, 75.34 ± 2.86, 73.99 ± 2.59 years)	Frailty	Body composition variables: whole-body phase angle, right leg lean mass, and ultrasound-measured muscle thickness
**Park et al. (2025) [52]**	South Korea	Cross-sectional study	To identify predictors of cognitive frailty (CF) and develop a predictive model using multidomain risk factors, Korean Frailty and Aging Cohort Study (KFACS)	Community-dwelling	2404 older adults aged 70–84 (CF 77.4 ± 3.8 vs. 75.4 ± 3.8 years)	Cognitive frailty (CF)	education, physical limitations, malnutritional status, balance
**Dong et al. (2025) [70]**	China	Longitudinal cohort study (based on public databases)	To develop and validate a risk prediction model for frailty in the elderly using a database from the Chinese Longitudinal Healthy Longevity Survey (CLHLS), 5-year follow-up	Community-dwelling	4878 participants aged ≥65 (87 years median)	Frailty (Frailty Index)	Age, BMI, cognitive function, gender, ethnicity, education.
**Tzeng et al. (2025) [53]**	Taiwan	Cross-sectional study	To explore how different components of the Falls Risk for Older People in the Community—Taiwan version (Tw-FROP-Com) relate to the five frailty phenotypes (unintentional weight loss, weakness, exhaustion, slow walking speed, and low physical activity) of Fried’s frailty criteria	Community-dwelling	375 participants aged ≥65 (75.39 ± 6.94 years)	Fried’s frailty: Fall risk	Age, education, BMI, comorbidities, ↓ scores on physical activity and gait speed, depression (exhaustion).

Note: ↑ indicates increased risk/value; ↓ indicates decreased risk/value.

**Table 3:** Categorization of identified factors associated with vulnerability and frailty.

Domain	Frequently Reported Factors (N of Studies)
Biological/Clinical	Chronological age (n = 26); Multimorbidity/co-morbidity (n = 15); Polypharmacy (n = 9); Uricemia (n = 2); Diabetes (n = 2); Ulcer risk (n = 2); Anticholinergic burden (n = 1).
Psychological	Depressive symptoms (n = 15); Cognitive impairment/dementia (n = 15); Poor self-rated health (n = 5); Sleep disturbances (n = 2).
Socioeconomic/Relational	Female gender (n = 14); Lower education (n = 7); Social isolation/low interaction (n = 7); Socioeconomic status/financial strain (n = 4); Living alone (n = 3); Marital status (unmarried/widowed) (n = 3); Race/ethnicity (n = 3); Infrequent visitation (n = 2).
Behavioral/Lifestyle	Physical inactivity (n = 4); Smoking history (n = 3); Alcohol intake (n = 2); Low health literacy (n = 1);
Self-care Profile	Functional dependence (ADL^1^ *-/IADL^2^ **-limitations) (n = 21); Objective mobility/strength decline (n = 10); Malnutrition/nutritional risk (n = 9); Body Mass Index abnormalities (n = 6); Fear of falling/fall history (n = 5); Swallowing difficulties (n = 3); Biomarkers (low albumin, anemia, HRR elevated CRP^3^ ***, plasma oxylipins); Oral health (n = 3) Pressure injury risk (n = 1); Chronic pain (n = 1).

1 * ADL = Activities of Daily Living, 2 ** IADL = Instrumental Activities of Daily Living/3 *** CRP = C-Reactive Protein. Values indicate the number of primary studies identifying each variable.

## Data Availability

All datasets generated and analyzed during this systematic review are included within this article, its main tables (Table 1, Table 2 and Table 3), and its appendixes ([Table A2, Table A3, Table A4, Table A5, Table A6, Table A7, Table A8 and Table A9]). The search queries and initial screening yields are publicly accessible via the registered PROSPERO record (CRD420251039796). Additional screening records and extraction sheets are available from the corresponding author upon reasonable request.

## Abbreviations

The following abbreviations are used in this manuscript:

a-ADL-DI	Advanced Activities of Daily Living–Disability Index
ADL	Activities of Daily Living
ALB	Serum Albumin
BMI	Body Mass Index
CCI	Charlson comorbidity index
CD-RISC	Connor–Davidson Resilience Scale
CF	Cognitive Frailty
CFS	Clinical Frailty Scale
CHF	Congestive Heart Failure
CKD	Chronic Kidney Disease
CLHLS	Chinese Longitudinal Healthy Longevity Survey
CRP	C-Reactive Protein
EFS	Edmonton Frail Scale
ELSA	English Longitudinal Study of Ageing
FI	Frailty Index
FREEDOM	French Acronym For Fragilité Et Evaluation à Domicile
HCBS	Home and Community-Based Services
HL	Health Literacy
HRR	Haemoglobin/Rdw Ratio
IADL	Instrumental Activities of Daily Living scale
JBI	Joanna Briggs Institute
K-ADL	Korean Activities of Daily Living scale
KCL	Kihon Checklist
KFACS	Korean Frailty and Aging Cohort Study
LASA	Longitudinal Aging Study Amsterdam
LPOPS	Living Profiles of Older People Survey
LSTM	Long Short-Term Memory
LWF	Living With Frailty
MUSIC	McMaster University Sentinel and Information Collaboration
MUST	Malnutrition Universal Screening Tool
PASE	Physical Activity Scale for the Elderly
PRISMA	Preferred Reporting Items for Systematic Reviews and Meta-Analyses
FP	Fried phenotype
SARC-F	Strength, Assistance with walking, Rising from chair, Climbing stairs, Falls
SARS-CoV-2	Severe Acute Respiratory Syndrome Coronavirus 2
SES	Socioeconomic Status
TFI	Tilburg frailty indicator
TSHA	Toledo Study on Healthy Ageing
TUG/TUGT	Timed Up and Go
Tw-FROP-	Falls Risk for Older People in the Community—Taiwan version
TxB2/7-HDoHE	Thromboxane B2/7-Hydroxydocosahexaenoic acid
WHO-5	WHO-5 Well-being Index

## Appendix A

**Table A1:** Full search syntax and database query parameters.

Database/Interface	Full Search Query/Advanced Syntax	Full Search Query/Advanced Syntax	Limits & Filters Applied	Initial Yield (n)
PubMed/MEDLINE	(“Vulnerable Populations”[Mesh] OR “Frail Elderly” [Mesh] OR “functionally impaired elderly”) AND (“Geriatric Assessment”[Mesh] OR “International Classification of Functioning, Disability and Health” [Mesh] OR “Health Status Indicators” [Mesh] OR “Quality of Life” [Mesh]) AND (prognosis OR predictor) NOT (review)	2 June 2025	Publication date: Last 5 years (2020–2025); Languages: English, French, Portuguese, Spanish.	466
Scopus (Elsevier)	TITLE-ABS-KEY ((“Vulnerable Populations” OR “Frail Elderly” OR “functionally impaired elderly”) AND (“Geriatric Assessment” OR “International Classification of Functioning, Disability and Health” OR “Health Status Indicators” OR “Quality of Life”) AND (prognosis OR predictor)) AND PUBYEAR > 2019 AND PUBYEAR < 2026 AND (LIMIT-TO (DOCTYPE, “ar”)) AND (LIMIT-TO (LANGUAGE, “Portuguese”) OR LIMIT-TO (LANGUAGE, “Spanish”) OR LIMIT-TO (LANGUAGE, “English”) OR LIMIT-TO (LANGUAGE, “French”))	3 June 2025	Publication years: 2020–2025; Document type: Article (“ar”); Languages: English, French, Portuguese, Spanish.	599
Web of Science (Clarivate)	(“Vulnerable Populations” OR “Frail Elderly” OR “functionally impaired elderly”) AND (“Geriatric Assessment” OR “International Classification of Functioning, Disability and Health” OR “Health Status Indicators” OR “Quality of Life”) AND (prognosis OR predictor)Query session: (https://www.webofscience.com/wos/woscc/summary/7df9cdc3-0dc7-4a76-86f4-1ff4400ac902-0165608ee8/relevance/1) (accessed on 4 June 2025)	4 June 2025	Timespan: 1 January 2020 to 29 May 2025; Document types: Articles; Languages: English, French, Portuguese, Spanish.	39
CINAHL (EBSCOhost)	(“Vulnerable Populations” OR “Frail Elderly” OR “functionally impaired elderly”) AND (“Geriatric Assessment” OR “International Classification of Functioning, Disability and Health” OR “Health Status Indicators” OR “Quality of Life”) AND (prognosis OR predictor)	4 June 2025	Published Date: 1 January 2020–29 May 2025; Languages: English, French, Portuguese, Spanish.	77
Subtotal (Databases)	Consolidated database search yield			1181
Google Scholar	allintitle: ((Vulnerable OR FRAIL OR risk) AND aged 65)	5 June 2025	Screened for non-indexed grey literature; first 100 consecutive records sorted by default relevance.	100
Total Identified	Cumulative records entered into PRISMA 2020 Flow Diagram			1281

Note: The complete unedited strategy document and search logs are permanently accessible in the prospective PROSPERO record (CRD420251039796) at: (https://www.crd.york.ac.uk/PROSPEROFILES/d54273f12a9efe9533ad20638d86ba83.pdf) (accessed on 25 August 2026).

## Appendix B

**Table A2:** Results of the methodological quality assessment for the JBI critical appraisal tool of analytical quasi-experimental studies.

JBI Critical Appraisal Tool of Analytical Quasi-Experimental Studies (Non-Randomized Experimental Studies)												
Study	Rev	1	2	3	4	5	6	7	8	9	Score	Level
Huang et al. (2023) [31]	AR	Y	Y	Y	N	N	N	Y	Y	Y	6/9(67%)	Moderate
	HP	Y	Y	Y	N	N	N	Y	Y	Y	6/9(67%)	Moderate

Y—Yes; N—No.

**Table A3:** Results of the methodological quality assessment for the JBI critical appraisal tool of analytical cross-sectional studies.

JBI Critical Appraisal Tool of Analytical Cross-Sectional Studies											
Study	Rev	1	2	3	4	5	6	7	8	Score	Level
Steinmeyer et al. (2020) [32]	AR	Y	Y	Y	Y	Y	Y	Y	Y	8/8 (100%)	High
	HP	Y	Y	Y	Y	Y	Y	Y	Y	8/8 (100%)	High
Venturini et al. (2021) [33]	AR	Y	Y	Y	Y	U	N	Y	Y	6/8 (75%)	Moderate
	HP	Y	Y	Y	Y	U	N	Y	Y	6/8 (75%)	Moderate
Sharma et al. (2021) [34]	AR	Y	Y	Y	Y	Y	Y	Y	Y	8/8 (100%)	High
	HP	Y	Y	Y	Y	Y	Y	Y	Y	8/8 (100%)	High
Jung et al. (2021) [35]	AR	Y	Y	Y	Y	Y	Y	Y	Y	8/8 (100%)	High
	HP	Y	Y	Y	Y	Y	Y	Y	Y	8/8 (100%)	High
Sella-Weiss et al. (2021) [36]	AR	Y	Y	Y	Y	Y	Y	Y	Y	8/8 (100%)	High
	HP	Y	Y	Y	Y	Y	Y	Y	Y	8/8 (100%)	High
Tavares et al. (2022) [37]	AR	Y	Y	Y	Y	Y	Y	Y	Y	8/8 (100%)	High
	HP	Y	Y	Y	Y	Y	Y	Y	Y	8/8 (100%)	High
Mangin et al. (2023) [38]	AR	Y	Y	Y	Y	N	N	Y	Y	6/8 (75%)	Moderate
	HP	Y	Y	Y	Y	N	N	Y	Y	6/8 (75%)	Moderate
Yan et al. (2023) [39]	AR	Y	Y	Y	Y	Y	Y	Y	Y	8/8 (100%)	High
	HP	Y	Y	Y	Y	Y	Y	Y	Y	8/8 (100%)	High
Zhu et al. (2023) [40]	AR	Y	Y	Y	Y	Y	Y	Y	Y	8/8 (100%)	High
	HP	Y	Y	Y	Y	Y	Y	Y	Y	8/8 (100%)	High
Shakya et al. (2023) [41]	AR	Y	Y	Y	Y	Y	Y	Y	Y	8/8 (100%)	High
	HP	Y	Y	Y	Y	Y	Y	Y	Y	8/8 (100%)	High
Cömertoğlu et al. (2023) [42]	AR	U	Y	Y	Y	Y	Y	Y	Y	7/8 (88%)	High
	HP	U	Y	Y	Y	Y	Y	Y	Y	7/8 (88%)	High
Henry et al. (2023) [43]	AR	Y	Y	Y	Y	Y	Y	Y	Y	8/8 (100%)	High
	HP	Y	Y	Y	Y	Y	Y	Y	Y	8/8 (100%)	High
Alamri & Mealif (2024) [44]	AR	Y	Y	Y	Y	Y	Y	Y	Y	8/8 (100%)	High
	HP	Y	Y	Y	Y	Y	Y	Y	Y	8/8 (100%)	High
Johnson et al. (2024) [45]	AR	Y	Y	Y	Y	Y	Y	Y	Y	8/8 (100%)	High
	HP	Y	Y	Y	Y	Y	Y	Y	Y	8/8 (100%)	High
Sepúlveda et al. (2024) [46]	AP	Y	Y	Y	Y	Y	Y	Y	Y	8/8 (100%)	High
	HP	Y	Y	Y	Y	Y	Y	Y	Y	8/8 (100%)	High
Güner et al. (2024) [47]	AR	Y	Y	Y	Y	Y	Y	Y	Y	8/8 (100%)	High
	HP	Y	Y	Y	Y	Y	Y	Y	Y	8/8 (100%)	High
Tang et al. (2024) [48]	AR	Y	Y	Y	Y	Y	Y	Y	Y	8/8 (100%)	High
	HP	Y	Y	Y	Y	Y	Y	Y	Y	8/8 (100%)	High
Savci et al. (2025) [49]	AR	Y	Y	Y	Y	Y	Y	Y	Y	8/8 (100%)	High
	HP	Y	Y	Y	Y	Y	Y	Y	Y	8/8 (100%)	High
Ceylan et al. (2025) [50]	AR	Y	Y	Y	Y	Y	Y	Y	Y	8/8 (100%)	High
	HP	Y	Y	Y	Y	Y	Y	Y	Y	8/8 (100%)	High
Ortiz-Navarro et al. (2025) [51]	AR	Y	Y	Y	Y	Y	Y	Y	Y	8/8 (100%)	High
	HP	Y	Y	Y	Y	Y	Y	Y	Y	8/8 (100%)	High
Park et al. (2025) [52]	AR	Y	Y	Y	Y	Y	Y	Y	Y	8/8 (100%)	High
	HP	Y	Y	Y	Y	Y	Y	Y	Y	8/8 (100%)	High
Tzeng et al. (2025) [53]	AR	Y	Y	Y	Y	Y	Y	Y	Y	8/8 (100%)	High
	HP	Y	Y	Y	Y	Y	Y	Y	Y	8/8 (100%)	High

Y—Yes; N—No; U—Unclear.

**Table A4:** Results of the methodological quality assessment for the JBI critical appraisal tool of analytical cohort studies.

JBI Critical Appraisal Tool of Analytical Cohort Studies														
Study	Rev	1	2	3	4	5	6	7	8	9	10	11	Score	Level
Hajek et al. (2020) [54]	AR	Y	Y	Y	Y	Y	N	Y	Y	N	N	Y	8/11 (73%)	Moderate
	HP	Y	Y	Y	Y	Y	N	Y	Y	N	N	Y	8/11 (73%)	Moderate
Lewis (2020) [55]	AR	Y	Y	Y	N *	N *	N	Y	Y	Y	N	Y	7/11 (64%)	Moderate
	HP	Y	Y	Y	N *	N *	N	Y	Y	Y	N	Y	7/11 (64%)	Moderate
Breij et al. (2020) [56]	AR	Y	Y	Y	Y	Y	N	Y	Y	N	N	Y	8/11 (73%)	Moderate
	HP	Y	Y	Y	Y	Y	N	Y	Y	N	N	Y	8/11 (73%)	Moderate
Varan et al. (2021) [57]	AR	Y	Y	Y	Y	Y	Y	Y	Y	Y	Y	U	10/11 (91%)	High
	HP	Y	Y	Y	Y	Y	Y	Y	Y	Y	Y	U	10/11 (91%)	High
Rodríguez-Laso et al. (2021) [58]	AR	Y	Y	Y	Y	Y	N	Y	Y	N	N	Y	8/11 (73%)	Moderate
	HP	Y	Y	Y	Y	Y	N	Y	Y	N	N	Y	8/11 (73%)	Moderate
Tsay et al. (2021) [59]	AR	Y	Y	Y	Y	Y	N	Y	Y	Y	N	Y	9/11 (82%)	High
	HP	Y	Y	Y	Y	Y	N	Y	Y	Y	N	Y	9/11 (82%)	High
Mielke et al. (2022) [60]	AR	Y	Y	Y	Y	Y	N	Y	Y	N	Y	Y	9/11 (82%)	High
	HP	Y	Y	Y	Y	Y	N	Y	Y	N	Y	Y	9/11 (82%)	High
Lee et al. (2022) [61]	AR	Y	Y	Y	Y	Y	Y	Y	Y	Y	Y	Y	11/11 (100%)	High
	HP	Y	Y	Y	Y	Y	Y	Y	Y	Y	Y	Y	11/11 (100%)	High
Thompson et al. (2022) [62]	AR	Y	Y	Y	Y	Y	N	Y	Y	Y	N	Y	9/11 (82%)	High
	HP	Y	Y	Y	Y	Y	N	Y	Y	Y	N	Y	9/11 (82%)	High
Yoshizawa (2022) [63]	AR	Y	Y	Y	Y	Y	Y	Y	Y	N	N	Y	9/11 (82%)	High
	HP	Y	Y	Y	Y	Y	Y	Y	Y	N	N	Y	9/11 (82%)	High
Rodríguez-Laso et al. (2023) [64]	AR	Y	Y	Y	Y	Y	N	Y	Y	N	Y	Y	9/11 (82%)	High
	HP	Y	Y	Y	Y	Y	N	Y	Y	N	Y	Y	9/11 (82%)	High
Costenoble et al. (2023) [65]	AR	Y	Y	Y	Y	Y	Y	Y	N	Y	N	Y	9/11 (82%)	High
	HP	Y	Y	Y	Y	Y	Y	Y	N	Y	N	Y	9/11 (82%)	High
Tchalla et al. (2023) [66]	AR	Y	Y	Y	Y	Y	N	Y	Y	Y	N	Y	9/11 (82%)	High
	HP	Y	Y	Y	Y	Y	N	Y	Y	Y	N	Y	9/11 (82%)	High
Van der Ploeg et al. (2024) [67]	AR	Y	Y	Y	Y	Y	N	Y	Y	N	N	Y	8/11 (73%)	Moderate
	HP	Y	Y	Y	Y	Y	N	Y	Y	N	N	Y	8/11 (73%)	Moderate
Suprawesta et al. (2024) [68]	AR	Y	Y	Y	Y	Y	N	Y	Y	N	Y	Y	9/11 (82%)	High
	HP	Y	Y	Y	Y	Y	N	Y	Y	N	Y	Y	9/11 (82%)	High
Tanaka et al. (2025) [69]	AR	Y	Y	Y	Y	Y	Y	Y	Y	N	Y	Y	10/11 (91%)	High
	HP	Y	Y	Y	Y	Y	Y	Y	Y	N	Y	Y	10/11 (91%)	High
Dong et al. (2025) [70]	AR	Y	Y	Y	Y	Y	Y	Y	Y	N	Y	Y	10/11 (91%)	High
	HP	Y	Y	Y	Y	Y	Y	Y	Y	N	Y	Y	10/11 (91%)	High

Y—Yes; N—No; U—Unclear. * Note on Consensus Adjudication: For Lewis (2020) [55], initial reviewer discordance on Items 4 and 5 (measurement of exposure and identification of confounding) was arbitrated with a third reviewer (A.C.); because exposure protocols in the virtual ward setting relied on administrative health provider counts and confounding adjustments were restricted to baseline univariable comparisons for certain sub-outcomes, Items 4 and 5 were adjudicated as ‘No’, confirming a final consensus score of 7/11 (64%, Moderate quality).

## Appendix C

**Table A5:** Master Study Identification and Cohort Mapping Table (*N* = 40).

ID	Author & Year & Reference	Full Article Title & Journal Citation	Persistent Identifier (DOI/PMID)	Underlying Cohort/Data Source	Study Design	Country & Setting	Sample Size (n)
1	Hajek et al. (2020) [54]	Which Factors Contribute to Frailty among the Oldest Old? Results of the AgeCoDe and AgeQualiDe Study. Gerontology, 66(5), 460–466.	DOI: 10.1159/000508723	AgeCoDe/AgeQualiDe	Cohort Study	Germany (Community-dwelling)	486
2	Lewis (2020) [55]	Risk Prediction for Adverse Outcomes for Frail Older Persons with Complex Healthcare and Social Care Needs Admitted to a Community Virtual Ward Model	DOI: 10.2147/CIA.S236895	Community Virtual Ward (CVW) Cohort	Cohort Study	Ireland (Community-dwelling)	88
3	Steinmeyer et al. (2020) [32]	Hemoglobin concentration; a pathway to frailty. BMC Geriatrics, 20, 203.	DOI: 10.1186/s12877-020-01597-6	Toulouse University Hospital Geriatric Day Hospital Cohort	Cross-sectional Study	France (Hospital)	1829
4	De Breij et al. (2020) [56]	Predictors of Frailty and Vitality in Older Adults Aged 75 years and Over: Results from the Longitudinal Aging Study Amsterdam. Gerontology, 67(1), 69–77.	DOI: 10.1159/000512049	Longitudinal Aging Study Amsterdam (LASA)	Cohort Study	Netherlands (Community-dwelling)	569
5	Venturini et al. (2021) [33]	A multidimensional approach to frailty in Brazilian older adults: the FIBRA-BR study. BMC Geriatrics, 21, 143.	DOI: 10.1186/s12877-021-02193-y	FIBRA-BR Study (Frailty in Brazilian Older People)	Cross-sectional Study	Brazil (Community-dwelling)	3569
6	Varan et al. (2021) [57]	Comparative Evaluation of Predictive Ability of Comprehensive Geriatric Assessment Components Including Frailty on Long-Term Mortality	DOI: 10.1080/0361073X.2021.1876395	Gazi University Geriatric Outpatient Clinic Cohort	Cohort Study	Turkey (Community-dwelling)	967
7	Sharma et al. (2021) [34]	Nutritional risk assessed by the Malnutrition Universal Screening Tool as a predictor of frailty in acutely hospitalised older patients: An observational study. Asia Pacific Journal of Clinical Nutrition, 30(2), 187–195.	DOI: 10.6133/apjcn.202106_30(2).0001	Flinders Medical Centre Acute Inpatient Cohort	Cross-sectional Study	Australia (Hospital)	243
8	Jung et al. (2021) [35]	Frailty Assessment Using Temporal Gait Characteristics and a Long Short-Term Memory Network. IEEE Journal of Biomedical and Health Informatics	DOI: 10.1109/JBHI.2021.3067931	Kyung Hee University Medical Center Cohort	Cross-sectional Study	South Korea (Community-dwelling)	74
9	Sella-Weiss et al. (2021) [36]	Association between swallowing function, malnutrition and frailty in community dwelling older people. Clinical Nutrition ESPEN, 45, 476–485.	DOI: 10.1016/j.clnesp.2021.06.028	Haifa & Ono Academic College Community Cohort	Cross-sectional Study	Israel (Community-dwelling)	180
10	Rodríguez-Laso et al. (2021) [58]	Transitions Between Frailty States and Its Predictors in a Cohort of Community-Dwelling Spaniards. JAMDA, 23(4), 524.e1–524.e11.	DOI: 10.1016/j.jamda.2021.07.008	Toledo Study for Healthy Aging (TSHA)	Cohort Study	Spain (Community-dwelling)	1748
11	Tsay et al. (2021) [59]	Predictors of Frailty Change in Home and Community-Based Services Populations	DOI: 10.1016/j.jamda.2021.07.032	Connecticut Medicaid HCBS Database (interRAI-HC)	Cohort Study	USA (Community-dwelling)	16,309
12	Tavares et al. (2022) [37]	Predictors of frailty in older people users of Primary Health Care. Revista Brasileira de Enfermagem	DOI: 10.1590/0034-7167-2020-1292	Vila Nova de Gaia Family Health Unit (FHU) Cohort	Cross-sectional Study	Portugal (Community-dwelling)	136
13	Mielke et al. (2022) [60]	Gender differences in frailty transition and its prediction in community-dwelling old adults. Scientific Reports	DOI: 10.1038/s41598-022-11358-7	Berlin Initiative Study (BIS)	Cohort Study	Germany (Community-dwelling)	1158
14	Lee et al. (2022) [61]	The influence of multiple frailty profiles on institutionalization and all-cause mortality in community-living older adults. Journal of Cachexia, Sarcopenia and Muscle, 13(5), 2322–2330.	DOI: 10.1002/jcsm.13033	Living Profiles of Older People Survey (LPOPS)	Cohort Study	South Korea (Community-dwelling)	9171
15	Thompson et al. (2022) [62]	Frailty change based on minimally important difference in nursing home residents: FIRST cohort study findings. Age and Ageing, 51(5), afac246.	DOI: 10.1093/ageing/afac246	FIRST Cohort Study (Frailty in Residential Sector Teams)	Cohort Study	Australia (Long-term care/nursing home)	548
16	Yoshizawa (2022) [63]	Impact of Health Literacy on the Progression of Frailty after 4 Years among Community-Dwelling Older Adults. IJERPH, 19(1), 394.	DOI: 10.3390/ijerph19010394	Kashiwa Longitudinal Study	Cohort Study	Japan (Community-dwelling)	621
17	Rodríguez-Laso et al. (2023) [64]	Predictors of Maintained Transitions Between Robustness and Prefrailty in Community-Dwelling Older Spaniards. JAMDA, 24(1), 57–64.	DOI: 10.1016/j.jamda.2022.10.010	Toledo Study for Healthy Aging (TSHA)	Cohort Study	Spain (Community-dwelling)	949
18	Mangin et al. (2023) [38]	Association between frailty, chronic conditions and socioeconomic status in community-dwelling older adults attending primary care: a cross-sectional study using practice-based research network data. BMJ Open	DOI: 10.1136/bmjopen-2022-066269	MUSIC PBRN (McMaster University Practice-Based Network)	Cross-sectional Study	Canada (Community-dwelling)	2043
19	Yan et al. (2023) [39]	Analysis of systolic and diastolic blood pressure and frailty in community-dwelling older adults. Medicine	DOI: 10.1097/MD.0000000000032874	Nantong University Affiliated Hospital Geriatric Cohort	Cross-sectional Study	China (Hospital)	150
20	Huang et al. (2023) [31]	Physical activity plays a crucial role in multidomain intervention for frailty prevention. Aging Clinical and Experimental Research, 35(6), 1283–1292.	DOI: 10.1007/s40520-023-02412-z	Kaohsiung Community Care Centers Intervention Cohort	Quasi-experimental Study	Taiwan (Kaohsiung, Community Centers)	106
21	Zhu et al. (2023) [40]	Lower haemoglobin-to-red blood cell distribution width ratio is independently associated with frailty in community-dwelling older adults: a cross-sectional study. BMJ Open.	DOI: 10.1136/bmjopen-2022-069141	Wuhan Community Older Adult Health Study	Cross-sectional Study	China (Community-dwelling)	1296
22	Costenoble et al. (2023) [65]	Transitions in robust and prefrail octogenarians after 1 year: the influence of activities of daily living, social participation, and psychological resilience on the frailty state. BMC Geriatrics, 23, 485.	DOI: 10.1186/s12877-023-04178-5	BUTTERFLY Study (Vrije Universiteit Brussel)	Cohort Study	Belgium (Community-dwelling)	322
23	Shakya et al. (2023) [41]	Does cumulative psychosocial stress explain frailty disparities in community-dwelling older adults? Archives of Gerontology and Geriatrics, 113, 105055.	DOI: 10.1016/j.archger.2023.105055	Health and Retirement Study (HRS)	Cross-sectional Study	USA (Community-dwelling)	7679
24	Tchalla et al. (2023) [66]	Patterns, predictors, and outcomes of frailty trajectories in community-dwelling older adults: Results from The FREEDOM Cohort Study. Archives of Gerontology and Geriatrics, 113, 105080.	DOI: 10.1016/j.archger.2023.105101	FREEDOM Cohort Study	Cohort Study	France (Community-dwelling)	497
25	Cömertoğlu et al. (2023) [42]	The Effect of Social Frailty on Mental Health and Quality of Life in Older People: A Cross-Sectional Study. Research Square.	DOI: 10.21203/rs.3.rs-2852088/v1	Hacettepe University Geriatric Outpatient Cohort	Cross-sectional Study	Turkey (Community-dwelling)	136
26	Henry et al. (2023) [43]	Social Frailty in Late Adulthood: Social Cognitive and Psychological Well-Being Correlates. The gerontological society of america.	DOI: 10.1093/geronb/gbac157	Brisbane Community Older Adult Survey	Cross-sectional Study	Australia (Community-dwelling)	90
27	Van der Ploeg et al. (2024) [67]	Disability transitions in Dutch community-dwelling older people aged 75 years or older. Archives of Gerontology and Geriatrics, 116, 105165.	DOI: 10.1016/j.archger.2023.105165	Roosendaal Longitudinal Study	Cohort Study	Netherlands (Community-dwelling)	479
28	Suprawesta et al. (2024) [68]	Factors affecting cognitive frailty improvement and progression in Taiwanese older adults. BMC Geriatrics, 24, 105.	DOI: 10.1186/s12877-024-04700-3	Taipei Medical University Outpatient & Community Cohort	Cohort Study	Taiwan (Community-dwelling)	832
29	Alamri & Mealif (2024) [44]	Exploring the correlates of frailty among hospitalized older adults: A cross-sectional study in a Saudi teaching hospital. Medicine, 103(25), e38603.	DOI: 10.1097/MD.0000000000038603	King Abdulaziz University Hospital (KAUH) Inpatient Database	Cross-sectional Study	Saudi Arabia (Hospital)	147
30	Johnson et al. (2024) [45]	Frailty or frailties: exploring frailty index subdimensions in the English Longitudinal Study of Ageing. JECH, 78(10), 609–115.	DOI: 10.1136/jech-2023-221829	English Longitudinal Study of Ageing (ELSA, Wave 9)	Cross-sectional Study	UK (Community-dwelling)	4971
31	Sepúlveda et al. (2024) [46]	Physiological changes associated with aging: Identification of novel biomarkers for frailty syndrome in women. Free Radical Biology and Medicine, 223, 160–171.	DOI: 10.1016/j.freeradbiomed.2024.07.022	PIEI-ES Healthy Aging Program (Talca/San Clemente)	Cross-sectional Study	Chile (Community-dwelling)	71
32	Güner et al. (2024) [47]	A hand-in-hand phenomenon in older adults: Increased risk of frailty in geriatric outpatients associated with handgrip strength asymmetry and weakness. Clinical Nutrition, 43(10), 2381–2387.	DOI: 10.1016/j.clnu.2024.09.010	Hacettepe University Geriatric Medicine Outpatient Cohort	Cross-sectional Study	Turkey (Community-dwelling)	512
33	Tang et al. (2024) [48]	Development of a Frailty Prediction Model Among Older Adults in China: A Cross-Sectional Analysis Using the Chinese Longitudinal Healthy Longevity Survey. Nursing Open, 11(4), e70070.	DOI: 10.1002/nop2.70070	Chinese Longitudinal Healthy Longevity Survey (CLHLS 2018 Wave)	Cross-sectional Study	China (Community-dwelling)	9006
34	Savci et al. (2025) [49]	Relationship between frailty, nutritional status, and physical performance in community-dwelling older adults. Archives of Gerontology and Geriatrics	DOI: 10.1016/j.gerinurse.2025.02.001	Community Health Care Centers Cohort	Cross-sectional Study	Turkey (Long-term care/nursing home)	205
35	Tanaka et al. (2025) [69]	Anticholinergic burden quantified using the Japanese risk scale as a predictor of frailty and sarcopenia among community-dwelling older adults: A 9-year Kashiwa cohort study. Geriatrics & Gerontology International, 25, 520–527.	DOI: 10.1111/ggi.70012	Kashiwa Longitudinal Population-Based Cohort Study	Cohort Study	Japan (Community-dwelling)	1549
36	Ceylan et al. (2025) [50]	Can handgrip strength alone detect individuals living with frailty according to the Clinical Frailty Scale?	DOI: 10.1590/1806-9282.20240237	Hacettepe University Geriatric Outpatient Cohort	Cross-sectional Study	Turkey (Community-dwelling)	742
37	Ortiz-Navarro et al. (2025) [51]	Identification of Target Body Composition Parameters by DXA, Bioelectrical Impedance, and Ultrasonography to Detect Older Adults With Frailty Using a Mobile App in Primary Care. JMIR Aging, 8, e67982.	DOI: 10.2196/67982	Andalusian Public Health System Primary Care Cohort	Cross-sectional Study	Spain (Community-dwelling)	94
38	Park et al. (2025) [52]	Predicting cognitive frailty in community-dwelling older adults: a machine learning approach based on multidomain risk factors. Scientific Reports.	DOI:10.1038/s41598-025-00844-3	Korean Frailty and Aging Cohort Study (KFACS)	Cross-sectional Study	South Korea (Community-dwelling)	2404
39	Dong et al. (2025) [70]	Development and validation of a prediction model of frailty risk in community-dwelling older adults: From a national longitudinal survey. Public Health, 240, 63–70.	DOI: 10.1016/j.puhe.2024.12.055	Chinese Longitudinal Healthy Longevity Survey (CLHLS)	Cohort Study	China (Community-dwelling)	4878
40	Tzeng et al. (2025) [53]	Examining frailty phenotypes of community-dwelling older adults in Taiwan using the falls risk for older people in the community—Taiwan version (Tw-FROP-Com). Aging, 17, 206231.	DOI: 10.18632/aging.206231	Taiwan Community Frailty Assessment Survey	Cross-sectional Study	Taiwan (Community-dwelling)	375
—	Cumulative Total						77,229

**Table A6:** Effect Estimates and Adjustment Models across Epidemiological Evidence Streams.

Study (Author, Year)	Setting/Cohort	Follow-Up	Exposure/Predictor	Outcome/Endpoint	Adjusted Effect Estimate	95% CI	Adjusted Covariates
	Stream 1: Concurrent Cross-Sectional Associations						
Steinmeyer et al. (2020) [32]	France (Outpatient Day Hospital)	N/A	Low Hemoglobin (<12 g/dL women, <13 men)	Gerontopôle Frailty Screening Tool (GFST)	OR = 0.86 per g/dL increase	0.79–0.94	Age, sex, eGFR, CRP, MMSE, MNA, living situation
Venturini et al. (2021) [33]	Brazil (Community, 15 cities)	N/A	Poor SRH, incontinence, depressive symptoms, no visits	Fried Frailty Phenotype (FIBRA-BR)	Factor 1 Phys: *p* < 0.001; Factor 3 Psych: *p* < 0.001	N/A (Factor scores)	Age, sex, sociodemographic factors
Sharma et al. (2021) [34]	Australia (Acute Hospital Wards)	N/A	MUST score ≥ 1 (Medium/High Risk)	Edmonton Frail Scale (EFS)	OR = 3.82 (High vs. Low risk)	1.74–8.38	Age, sex, comorbidity, polypharmacy, mobility
Jung et al. (2021) [35]	South Korea (Community)	N/A	Temporal gait variability (LSTM wearable sensors)	FRAIL Scale	Accuracy = 88.5%; Gait asymmetry *p* < 0.01	N/A (ML classifier)	Age, sex, body height
Sella-Weiss et al. (2021) [36]	Israel (Community)	N/A	H-EAT-10 score (Dysphagia risk)	FRAIL Scale & Grip Strength	β = 0.057 (Linear regression)	0.004–0.111	Age, MNA-SF score, Geriatric Depression Scale (GDS-SF)
Tavares et al. (2022) [37]	Portugal (Primary Care)	N/A	Age (years) & Physical capacity score	Fried Frailty Phenotype	OR = 1.11 per year; OR = 0.67 per capacity unit	1.03–1.20; 0.51–0.89	Age, sex, self-rated health, living conditions
Mangin et al. (2023) [38]	Canada (Primary Care PBRN)	N/A	≥5 chronic conditions & Neighborhood income decile	Clinical Frailty Scale (CFS)	χ^2^ = 137.92 (Multimorbidity); χ^2^ = 61.42 (SES)	N/A (Bivariate χ^2^)	Retained in unadjusted/bivariate stratification
Yan et al. (2023) [39]	China (Hospital Inpatient)	N/A	24h SBP/DBP variability & Low Albumin (<35 g/L)	Fried Frailty Phenotype	Albumin: *p* = 0.002; SBP variability: *p* = 0.014	N/A (ANOVA/Spearman)	Age, sex, BMI, fasting blood glucose
Zhu et al. (2023) [40]	China (Community)	N/A	Lower HRR (<9.97 cutoff)	Fried Frailty Phenotype	AUC = 0.802; OR = 3.42 (Low vs. Normal HRR)	2.11–5.54	Age, sex, BMI, SBP, DBP, fasting glucose, lipids
Shakya et al. (2023) [41]	USA (Nationwide HRS)	N/A	Cumulative stress index (0–6) & Minority status	Fried Frailty Phenotype	aOR = 1.50 per stress unit	1.32–1.71	Age, sex, income, education, chronic conditions
Cömertoğlu et al. (2023) [42]	Turkey (Geriatric Outpatient)	N/A	HALFT Scale (Social frailty score ≥ 2)	Clinical Frailty Scale & FRAIL	r = 0.428 (CFS correlation)	N/A (Correlation)	GDS, MMSE, ADL, IADL
Henry et al. (2023) [43]	Australia (Community)	N/A	Inappropriate social behavior & Demoralization	Physical & Cognitive Frailty	β = 0.24 (Inappropriate behavior)	0.04–0.44	Age, sex, depression, physical frailty, cognitive frailty
Alamri & Mealif (2024) [44]	Saudi Arabia (Hospital Inpatient)	N/A	Comorbidity index & Polypharmacy (≥5 drugs)	Clinical Frailty Scale (CFS)	OR = 1.49 per comorbidity unit; Polypharmacy *p* = 0.003	1.14–1.99	Height, comorbidity index, heart failure
Johnson et al. (2024) [45]	UK (Nationwide ELSA Wave 9)	N/A	Mobility, Daily Activities, Mental Health, Orientation	Frailty Index (58 items)	Factor 1 (Mobility): r = 0.929; Factor 3 (Mental): r = 0.512	N/A (Factor analysis)	Age, sex, CASP-19 quality of life score
Sepúlveda et al. (2024) [46]	Chile (Community)	N/A	TxB2 & 7-HDoHE plasma levels	Edmonton Frail Scale (EFS)	*p* = 0.012 (Oxylipin divergence)	N/A (Biomarker comparison)	Age, sex, cardiovascular risk factors
Güner et al. (2024) [47]	Turkey (Geriatric Outpatient)	N/A	Asymmetric HGS (>10% diff) & Weakness	Clinical Frailty Scale (CFS)	aOR = 2.85 (Asymmetry + Weakness)	1.62–5.01	Age, sex, BMI, MNA-SF, MMSE, GDS, polypharmacy
Tang et al. (2024) [48]	China (Nationwide CLHLS 2018)	N/A	Rural residence, education, sleep, exercise	Frailty Index (38 items, cutoff ≥ 0.25)	AUC = 0.831; Age 80–94 OR = 4.58; Exercise OR = 0.76	3.88–5.42; 0.63–0.92	Age, sex, residence, living status, education, BMI, sleep, smoking, alcohol
Savci et al. (2025) [49]	Turkey (Nursing Home Residents)	N/A	EAT-10 score ≥ 3 & GDS score ≥ 5	Edmonton Frail Scale (EFS)	r = 0.384 (Dysphagia vs. Frailty); r = 0.412 (GDS)	N/A (Correlation)	Age, sex, comorbidity count, medication count
Ceylan et al. (2025) [50]	Turkey (Geriatric Outpatient)	N/A	HGS < 20.5 kg (women), <31.5 kg (men)	Clinical Frailty Scale (CFS ≥ 4)	aOR = 2.11 (Female sex); aOR = 0.88 per kg HGS	1.34–3.32; 0.84–0.92	Age, sex, HGS, education, multimorbidity, polypharmacy
Ortiz-Navarro et al. (2025) [51]	Spain (Primary Care)	N/A	Relative muscle power (RPOW) & Rectus femoris US	Fried Phenotype & SPPB	AUC = 0.892 (RPOW); US thickness *p* = 0.004	0.812–0.972	Age, sex, BMI, MNA, GPCOG, 5STS, 4 m walk speed
Park et al. (2025) [52]	South Korea (Nationwide KFACS)	N/A	Sensor-based STS velocity & Cognitive impairment	Cognitive Frailty (PF + IC)	aOR = 4.71 (Slowness); aOR = 5.09 (Weakness)	3.74–5.93; 3.98–6.51	Age, sex, education, comorbidities, prescription count, GDS
Tzeng et al. (2025) [53]	Taiwan (Community)	N/A	Fall history & 5 Fried Phenotype items	Fried Phenotype & Fall Risk Score	aOR = 15.51 (Frail vs. Non-frail on Fall Risk)	4.53–53.15	Age, education, BMI, comorbidities, physical activity, gait speed
	Stream 2: Baseline Determinants Predicting Frailty Onset						
Hajek et al. (2020) [54]	Germany (AgeCoDe/AgeQualiDe)	20 months	Age ≥ 85 years; Oldest-old primary care	Clinical Frailty Scale (CFS)	Fixed Effects β = 0.14 per age year	0.05–0.23	Panel fixed-effects controlling all time-invariant unobserved heterogeneity
Lewis (2020) [55]	Ireland (Community Virtual Ward)	90 days	>7 HCPs in Red CVW; <2 care episodes	Rockwood Clinical Frailty Scale (CFS)	OR = 14.77 (>7 HCPs); OR = 44.89 (<2 care episodes)	2.69–81.2; 12.4–162.1	Age, sex, comorbidity index, baseline CFS, home care hours
Breij et al. (2020) [56]	Netherlands (LASA Cohort)	3 years	Polypharmacy (≥5 drugs), MMSE, GDS	LASA Frailty Index (FI ≥ 0.25)	aOR = 2.14 (Polypharmacy); OR = 1.33 (Pain)	1.53–4.19; 1.18–1.71	Age, sex, depressive symptoms, chronic diseases, self-rated health
Varan et al. (2021) [57]	Turkey (Gazi Geriatric Outpatient)	47 months (median)	Albumin < 3.5 g/dL; 15ft walking time > 8.9 s	Edmonton Frail Scale (EFS) & Fried Phenotype	HR = 0.68 per g/dL Albumin; HR = 1.12 per sec walking	0.52–0.89; 1.05–1.21	Age, sex, comorbidity count, ADL, IADL, MMSE
Rodríguez-Laso et al. (2021) [58]	Spain (Toledo Study TSHA Wave 1–2)	5.2 years (median)	Fried phenotype items & lifestyle factors	Fried Frailty Phenotype (Transitions)	OR = 1.10 per age year; OR = 1.12 per GDS point	1.06–1.15; 1.01–1.24	Age, sex, education, BMI, alcohol, PASE, IADL, gait speed, grip strength
Tsay et al. (2021) [59]	USA (Connecticut Medicaid HCBS)	8–16 months	In-home care vs. Adult day services received	interRAI-HC Frailty Index	OR = 1.48 (ADL decline); OR = 0.68 (In-home care improvement)	1.03–2.12; 0.47–0.97	Age, sex, race/ethnicity, living alone, baseline frailty tier
Mielke et al. (2022) [60]	Germany (Berlin Initiative Study BIS)	2.1 years	Cognitive impairment, CHF, Diabetes, ACR	Fried Frailty Phenotype	Women Worsening OR = 3.70 (Cognitive); Men Worsening OR = 2.43	1.73–7.92; 1.20–4.93	Age, self-rated health, CHF, ACR, GFR, stroke, diabetes, living alone, smoking
Lee et al. (2022) [61]	South Korea (LPOPS Nationwide)	3 years	Single vs. 2-, 3-, or 4-domain frailty profiles	Institutionalization & All-Cause Mortality	Mixed Frailty (4 domains) HR = 3.89 (Mortality)	2.57–5.85	Age, sex, smoking, alcohol drinking, comorbidities
Thompson et al. (2022) [62]	Australia (FIRST Cohort Residential)	12 months	MNA At Risk/Malnourished vs. Normal	47-item Frailty Index (MID change)	OR = 1.88 (At Risk); OR = 2.02 (Malnourished)	1.28–2.75; 1.17–3.48	Age, sex, length of stay, MI, stroke, COPD, dementia, diabetes, polypharmacy
Yoshizawa (2022) [63]	Japan (Kashiwa Study)	4 years	Limited vs. High Health Literacy	Kihon Checklist (KCL)	aOR = 1.85 (Limited Health Literacy)	1.18–2.91	Age, sex, education, living alone, baseline KCL score, comorbidities
Rodríguez-Laso et al. (2023) [64]	Spain (Toledo Study TSHA Wave 1–3)	8 years (6.5–9.5 years)	Number of medications (continuous) & Low grip strength	Fried Phenotype (Persistent Transitions)	OR = 0.92 per additional drug; OR = 0.80 per age year	0.86–0.99; 0.72–0.89	Age, sex, alcohol consumption, chair stands time, grip strength, uricemia
Costenoble et al. (2023) [65]	Belgium (BUTTERFLY Cohort)	1 year	Brussels Integrated ADL Inventory (a-ADL-DI)	Adapted Fried Frailty Phenotype	a-ADL Worsening OR = 1.048; Women Improvement OR = 3.682	1.01–1.09; 1.38–10.14	Age, sex, b-ADL, i-ADL, board membership change, resilience score
Tchalla et al. (2023) [66]	France (FREEDOM Cohort)	6 years (75 months)	GDS > 9 & MMS < 24	Fried Phenotype (Group-based Trajectories)	OR = 10.51 (Depressive symptoms); OR = 4.56 (MMS < 24)	4.61–23.95; 1.97–10.58	Age, sex, hypertension, MMS, GDS, personalized care plan
Van der Ploeg et al. (2024) [67]	Netherlands (Roosendaal Cohort)	9 years	≥2 chronic diseases & Unhealthy lifestyle (TFI)	Groningen Activity Restriction Scale (GARS)	GEE β = 5.3 (Multimorbidity); β = 6.6 (Unhealthy lifestyle)	*p* = 0.002; *p* = 0.029	Age, sex, marital status, education, monthly income, life events
Suprawesta et al. (2024) [68]	Taiwan (Taipei Community)	2 years	Tinetti balance score, gait velocity, exercise habit	Cognitive Frailty Trajectories (GBTM)	OR = 0.58 (Regular exercise); OR = 2.90 (≥4 comorbidities)	0.36–0.94; 1.65–5.10	Age, sex, Tinetti balance, gait velocity, comorbidities, GDS score
Tanaka et al. (2025) [69]	Japan (Kashiwa 9-year Cohort)	9 years (6.0 years median)	Japan ACB score ≥ 3 vs. 0	CHS Index (Incident Frailty) & AWGS (Sarcopenia)	Adjusted HR = 2.45 (Frailty); Adjusted HR = 2.01 (Sarcopenia)	1.52–3.94; 1.20–3.35	Age, sex, BMI, education, income, living alone, cognitive, depression, food diversity, exercise, alcohol, comorbidities, polypharmacy, PIMs
Dong et al. (2025) [70]	China (Nationwide CLHLS 3 Cohorts)	9–10 years (65 mo median)	Cox stepwise selected 9-variable panel	Frailty Index (46 health deficits, cutoff ≥0.25)	AUC = 0.80 at 96 mo; High Risk PI *p* < 0.0001	0.78–0.82	Age, logBMI, MMSE, sex, ethnicity, education, occupation, smoking, natural teeth
	Stream 3: Baseline Frailty Predicting Downstream Outcomes						
Lewis (2020) [55]	Ireland (CVW Cohort)	90 days	Rockwood CFS (Red/Amber/Green Wards)	Institutionalization/Unplanned Hospital Admission	OR = 14.77 (Red Ward > 7 HCPs); OR = 9.43 (Unstable state)	2.69–81.2; 1.82–48.9	Age, comorbidity index, baseline CFS, care hours, FRIDs
Varan et al. (2021) [57]	Turkey (Gazi Outpatient)	47 months (median)	Edmonton Frail Scale (EFS ≥ 7) & Fried Phenotype	All-Cause Mortality	HR = 2.84 (EFS Frail vs. Robust); HR = 2.12 (Fried Frail)	1.65–4.88; 1.34–3.35	Age, sex, comorbidity count, serum albumin, IADL, walking time
Lee et al. (2022) [61]	South Korea (LPOPS Cohort)	3 years	Multidimensional Frailty Profiles (Physical, Cog, Psych, Soc)	Nursing Home Institutionalization & All-Cause Mortality	SHR = 3.42 (Institutionalization); HR = 3.89 (Mortality for Mixed Frailty)	1.79–6.52; 2.57–5.85	Age, sex, smoking, alcohol drinking, comorbidities
Thompson et al. (2022) [62]	Australia (FIRST Cohort Nursing Home)	1 year	47-item Frailty Index (Continuous deciles)	12-month All-Cause Mortality	HR = 2.15 per FI decile increment	1.42–3.25	Age, sex, length of stay, comorbidities, polypharmacy, MNA status
Mielke et al. (2022) [60]	Germany (BIS Cohort)	2.1 years	Fried Frailty Phenotype (Frail vs. Robust)	2.1-year All-Cause Mortality	33% mortality in Frail Men vs. 18% in Frail Women	*p* < 0.001 (Log-rank)	Age, sex, eGFR, Charlson Comorbidity Index, hospitalization history
Tchalla et al. (2023) [66]	France (FREEDOM Cohort)	6 years	Frailty Trajectory D (Severe Worsening)	Incident Falls & Unplanned Hospitalization	Falls: 43.8% in Traj D vs. 18.5% in Traj B; Hosp: 29.0%	*p* = 0.012 (Chi-squared across trajectories)	Age, sex, hypertension, MMS score, GDS score
Tanaka et al. (2025) [69]	Japan (Kashiwa Cohort)	9 years	Baseline Anticholinergic Burden (ACB ≥ 3) & Physical Frailty	Incident Sarcopenia & Long-Term Care Certification	Adjusted HR = 2.01 (Sarcopenia); HR = 2.45 (Frailty)	1.20–3.35; 1.52–3.94	Age, sex, BMI, education, income, living alone, cognitive, depression, exercise, comorbidities, polypharmacy, PIMs

Abbreviations: 5STS, Five-Times Sit-to-Stand test; a-ADL-DI, Advanced Activities of Daily Living Disability Index; ACB, Anticholinergic Cognitive Burden; ACR, Albumin-to-Creatinine Ratio; ADL, Activities of Daily Living; AgeCoDe, German Study on Ageing, Cognition and Dementia in Primary Care Patients; AgeQualiDe, follow-up study on needs, health service use, costs and health-related quality of life in the oldest old; aOR, adjusted Odds Ratio; AUC, Area Under the Curve; AWGS, Asian Working Group for Sarcopenia; b-ADL, Basic Activities of Daily Living; BIS, Berlin Initiative Study; BMI, Body Mass Index; BUTTERFLY, Brussels Study on the Early Predictors of Frailty; CASP-19, Control, Autonomy, Self-realization and Pleasure (19-item scale); CFS, Clinical Frailty Scale; CHF, Congestive Heart Failure; CHS, Cardiovascular Health Study; CI, Confidence Interval; CLHLS, Chinese Longitudinal Healthy Longevity Survey; COPD, Chronic Obstructive Pulmonary Disease; CRP, C-Reactive Protein; CSHA-FI, Canadian Study of Health and Aging Frailty Index; CVW, Community Virtual Ward; DBP, Diastolic Blood Pressure; EAT-10/H-EAT-10, (Hebrew) Eating Assessment Tool-10; eGFR, estimated Glomerular Filtration Rate; EFS, Edmonton Frail Scale; ELSA, English Longitudinal Study of Ageing; FI, Frailty Index; FIBRA-BR, Rede FIBRA (Frailty in Brazilian Elderly Network Study); FIRST, Frailty In Residential Sector over Time; FRAIL, Fatigue, Resistance, Ambulation, Illnesses, Loss of weight; FREEDOM, FRagilité Et Evaluation à DOMicile (Frailty and Evaluation at Home); FRIDs, Fall-Risk-Increasing Drugs; GARS, Groningen Activity Restriction Scale; GBTM, Group-Based Trajectory Modeling; GDS(-SF), Geriatric Depression Scale (-Short Form); GEE, Generalized Estimating Equations; GFR, Glomerular Filtration Rate; GFST, Gerontopôle Frailty Screening Tool; GPCOG, General Practitioner Assessment of Cognition; HALFT, social frailty scale (Help, Participation, Loneliness, Financial difficulty, Talk); HCBS, Home and Community-Based Services; HCPs, Health Care Professionals; HGS, Handgrip Strength; HR, Hazard Ratio; HRR, Hemoglobin-to-Red cell distribution width Ratio; HRS, Health and Retirement Study; i-ADL/IADL, Instrumental Activities of Daily Living; IC, Impaired Cognition; interRAI-HC, interRAI Home Care; KCL, Kihon Checklist; KFACS, Korean Frailty and Aging Cohort Study; LASA, Longitudinal Aging Study Amsterdam; LPOPS, Living Profiles of Older People Survey; LSTM, Long Short-Term Memory; MI, Myocardial Infarction; MID, Minimal/Minimally Important Difference; MMS/MMSE, Mini-Mental State (Examination); MNA(-SF), Mini Nutritional Assessment (-Short Form); MUST, Malnutrition Universal Screening Tool; N/A, Not Applicable; OR, Odds Ratio; PASE, Physical Activity Scale for the Elderly; PIMs, Potentially Inappropriate Medications; RPOW, Relative muscle Power; SBP, Systolic Blood Pressure; SES, Socioeconomic Status; SHR, Subdistribution Hazard Ratio; SPPB, Short Physical Performance Battery; SRH, Self-Rated Health; STS, Sit-To-Stand test; TFI, Tilburg Frailty Indicator; Traj, Trajectory; TSHA, Toledo Study for Healthy Aging; TxB2, Thromboxane B2; 7-HDoHE, 7-Hydroxydocosahexaenoic acid; US, Ultrasound.

**Table A7:** Distribution matrix of variables and health determinants identified in the studies included in the systematic review (n = 40).

Publication	Age	Functional Status/Physical Capacity/Exercise	Co-mobility	Depressive Symptoms/Sadness	Gender	Dementia/Memory Problems/Cognitive Status	Malnutrition or Risk Malnut.	Longer Walking Time/Asymmetry in Steps/Handstring Strength	Polypharmacy/Medication	Education	Social Interaction	BMI	Fear of Falling/Fall History	Poor Self-Rated Health	Marital Status	Smoke	Health Behaviors/Resilience	Socioeconomic Status	Living Alone	Alcohol	Swallowing	Hg or HRR (<9.97) or CRP	Race/Ethnic.	Oral Health/Teeth Status	Sleep	Uricemia	Low Albumin	Not visiting/Receiving	Diabetes	Pain	Ulcer risk	Anticholinergic Burden (Score ≥ 3)	Hospitalization	Long-Term Care	Biomarkers: txb2 and 7-hdohe	Low Health Literacy	High Blood Pressure (Or Bad Control)
Hajek et al. (2020) [54]	✔		✔			✔																															
Lewis (2020) [55]		✔				✔	✔																								✔						
Steinmeyer et al. (2020) [32]						✔	✔			✔								✔				✔															
Breij et al. (2020) [56]	✔		✔	✔	✔	✔			✔																					✔							
Venturini et al. (2021) [33]				✔		✔					✔		✔	✔										✔				✔									
Varan et al. (2021) [57]		✔			✔			✔																	✔		✔										
Sharma et al. (2021) [34]	✔		✔			✔	✔		✔			✔																									
Jung et al. (2021) [35]	✔				✔			✔																													
Sella-Weiss et al. (2021) [36]	✔			✔			✔	✔													✔																
Rodríguez-Laso et al. 2022 [58]	✔	✔		✔		✔		✔				✔				✔										✔											
Tsay et al. (2021) [59]	✔	✔									✔								✔				✔														
Tavares et al. (2022) [37]	✔	✔												✔																							
Mielke et al. (2022) [60]	✔		✔		✔	✔								✔					✔										✔				✔				
Lee et al. (2022) [61]	✔	✔	✔		✔						✔				✔		✔																				
Thompson et al. (2022) [62]	✔						✔																						✔								
Yoshizawa (2022) [63]	✔	✔		✔		✔					✔			✔				✔																		✔	
Rodríguez-Laso et al. (2023) [64]	✔	✔						✔	✔											✔						✔											
Mangin et al. (2023) [38]	✔		✔		✔													✔																			
Yan et al. (2023) [39]	✔		✔						✔																		✔										✔
Huang et al. (2023) [31]	✔	✔		✔									✔																								
Zhu et al. (2023) [40]																						✔															
Costenoble et al. (2023) [65]		✔									✔						✔																				
Shakya et al. (2023) [41]					✔					✔													✔														
Tchalla et al. (2023) [66]	✔		✔	✔		✔							✔																								
Cömertoğlu et al. (2023) [42]		✔		✔							✔				✔				✔									✔									
Henry et al. (2023) [43]				✔							✔																										
Van der Ploeg et al. (2024) [67]		✔	✔											✔																							
Suprawesta et al. (2024) [68]	✔	✔	✔	✔	✔																																
Alamri & Mealif (2024) [44]			✔						✔																												
Johnson et al. (2024) [45]		✔		✔		✔											✔																				
Sepúlveda et al. (2024) [46]																																			✔		
Güner et al. (2024) [47]	✔		✔		✔	✔	✔	✔	✔				✔																								
Tang et al. (2024) [48]	✔	✔			✔					✔		✔			✔	✔		✔		✔					✔									✔			
Savci et al. (2025) [49]	✔	✔		✔	✔																✔																
Tanaka et al. (2025) [69]	✔	✔	✔	✔		✔	✔	✔	✔			✔										✔										✔					
Ceylan et al. (2025) [50]	✔				✔	✔		✔	✔	✔																											
Ortiz-Navarro et al. (2025) [51]		✔						✔																													
Park et al. (2025) [52]	✔	✔	✔	✔	✔	✔	✔	✔	✔	✔											✔			✔													
Dong et al. (2025) [70]	✔	✔			✔					✔		✔				✔	✔						✔	✔													
Tzeng et al. (2025) [53]	✔	✔	✔	✔			✔			✔		✔	✔																								
Total	26	21	15	15	14	15	9	10	9	7	7	6	5	5	3	3	4	4	3	2	3	3	3	3	2	2	2	2	2	1	1	1	1	1	1	1	1

The checkmarks (✔) indicate that the variable was identified as a statistically significant factor or retained correlate/predictor in the corresponding study’s primary analytical model, with the final sum indicating the reporting frequency of each determinant across the selected literature (*N* = 40).

**Table A8:** Study-specific definitions, operational thresholds, and adjusted findings for medication and polypharmacy reports (n = 9).

Author & Year	Study Design & Setting	Medication Exposure Name	Specific Exposure Threshold/Definition	Outcome Measure	Follow-Up Duration	Adjusted Effect Estimate (OR/HR/β)	95% Confidence Interval	*p*-Value	Adjusted Covariates in Model	Reconciled Design Role
Sharma et al. (2021) [34]	Cross-sectional, Acute Hospital Wards	Polypharmacy	Concurrent use of ≥5 daily medications	Edmonton Frail Scale (EFS)	Cross-sectional (Admission)	Mean = 8.9 drugs (Frail) vs. 7.2 (Non-frail); *p* = 0.002	N/A (Mean diff)	0.002	Age, sex, MUST score, CCI, mobility, cognitive impairment	Concurrent Correlate (Stream 1)
Alamri & Mealif (2024) [44]	Cross-sectional, Hospital Inpatient Wards	Polypharmacy	Concurrent use of ≥5 daily medications	Clinical Frailty Scale (CFS)	Cross-sectional	Prevalence 47.6% (Frail) vs. 21.4% (Non-frail); *p* = 0.003	N/A (Chi-squared)	0.003	Height, comorbidity index, heart failure	Concurrent Correlate (Stream 1)
Ceylan et al. (2025) [50]	Cross-sectional, Geriatric Outpatient Clinic	Polypharmacy	Concurrent use of ≥5 daily medications	Clinical Frailty Scale (CFS ≥ 4)	Cross-sectional	71.3% (LWF) vs. 55.3% (Robust); *p* < 0.001	N/A (Univariate retained)	<0.001	Age, sex, HGS, education, multimorbidity	Concurrent Correlate (Stream 1)
Güner et al. (2024) [47]	Cross-sectional, Geriatric Outpatient Clinic	Polypharmacy	Concurrent use of ≥5 daily medications	Clinical Frailty Scale (CFS)	Cross-sectional	56.9% (Frail) vs. 45.7% (Non-frail); *p* = 0.015	N/A (Univariate retained)	0.015	Age, sex, BMI, MNA-SF, MMSE, GDS, HGS asymmetry	Concurrent Correlate (Stream 1)
De Breij et al. (2021) [56]	Longitudinal Prospective Cohort (LASA)	Polypharmacy	Concurrent use of ≥5 prescription drugs	LASA Frailty Index (FI ≥ 0.25)	3 years (2008–09 to 2011–12)	Adjusted aOR = 2.14 (39.6% baseline prevalence)	1.53–4.19	<0.001	Age, sex, depressive symptoms, chronic diseases, self-rated health, pain	Longitudinal Predictor (Stream 2)—Reconciled as Cohort
Rodríguez-Laso et al. (2023) [64]	Longitudinal Prospective Cohort (TSHA 3-Wave)	Continuous Medication Count	Total number of daily prescribed drugs (continuous)	Persistent Prefrailty to Robustness Transition	8 years (6.5–9.5 years)	Adjusted OR = 0.92 per additional drug	0.86–0.99	0.008	Age, grip strength, chair stands, drinking alcohol, uricemia	Longitudinal Predictor (Stream 2)
Tanaka et al. (2025) [69]	Longitudinal Prospective Cohort (Kashiwa)	Anticholinergic Burden & Polypharmacy	Japanese Anticholinergic Risk Scale (Score ≥ 3) & Polypharmacy (≥5 drugs)	Incident Physical Frailty & Sarcopenia	9 years (6.0 years median)	Adjusted HR = 2.45 (ACB ≥ 3 for Frailty); Adjusted HR = 2.01 (Sarcopenia)	1.52–3.94; 1.20–3.35	<0.001	Age, sex, BMI, education, income, living alone, cognitive, depression, food diversity, exercise, alcohol, comorbidities, polypharmacy, PIMs	Longitudinal Predictor (Stream 2)
Mielke et al. (2022) [60]	Longitudinal Prospective Cohort (BIS)	Polypharmacy	Concurrent use of ≥5 daily medications	Fried Frailty Phenotype (Worsening/Improvement)	2.1 years	73% baseline prevalence; retained in candidate predictor pool	N/A (Bootstrap selection)	N/S in final model	Age, self-rated health, cognitive impairment, CHF, stroke, diabetes, ACR, GFR, living alone, smoking	Longitudinal Predictor (Stream 2)
Lewis (2020) [55]	Prospective Correlational Cohort (CVW)	Medication Count & FRIDs	Mean medication count (8.24 SD 3.66) & Fall Risk Increasing Drugs (≥2 FRIDs)	Virtual Ward Risk Stratification/Unstable State	90 days	42.0% taking ≥ 2 FRIDs; Beta-blockers 32.9%, ACEi 26.1%	N/A (Baseline profile)	<0.05	Age, sex, Charlson Comorbidity Index, baseline CFS, home care hours	Longitudinal Cohort (Stream 2)

Note: Stream 1 denotes concurrent cross-sectional correlates; Stream 2 denotes baseline determinants prospectively predicting frailty onset, state progression, or transitions over longitudinal follow-up. Abbreviations: ACB, Anticholinergic Cognitive Burden; ACEi, Angiotensin Converting Enzyme inhibitors; ACR, Albumin-to-Creatinine Ratio; BIS, Berlin Initiative Study; BMI, Body Mass Index; CCI, Charlson Comorbidity Index; CFS, Clinical Frailty Scale; CHF, Congestive Heart Failure; CI, Confidence Interval; CVW, Community Virtual Ward; EFS, Edmonton Frail Scale; FI, Frailty Index; FRIDs, Fall-Risk-Increasing Drugs; GDS, Geriatric Depression Scale; GFR, Glomerular Filtration Rate; HGS, Handgrip Strength; HR, Hazard Ratio; LASA, Longitudinal Aging Study Amsterdam; LWF, Living With Frailty; MMSE, Mini-Mental State Examination; MNA-SF, Mini Nutritional Assessment—Short Form; MUST, Malnutrition Universal Screening Tool; N/A, Not Applicable; N/S, Not Significant; OR, Odds Ratio; PIMs, Potentially Inappropriate Medications; SD, Standard Deviation; TSHA, Toledo Study for Healthy Aging.

## Appendix D

**Table A9:** Bibliographic details, identifiers, and specific reasons for non-retrieval of full-text reports (n = 3).

ID	Bibliographic Details (DOI)	Base	Identifiers	Specific Reasons
UR-01	Vafaei, A., Wu, Y.Y., Curcio, C.L., Gomes, C.S., Auais, M., & Gomez, F. (2023). A Regression Tree Analysis to Identify Factors Predicting Frailty: The International Mobility in Aging Study. Gerontology, 69(2), 130–139. DOI: 10.1159/000526737.	Scopus/Web of Science	Community-dwelling older adults (≥65 years)	Full text unavailable through institutional library subscriptions or interlibrary loan networks; no response from the corresponding author following electronic inquiry.
UR-02	Kulal, N., Nandakumar, G., & Vaishali, K. (2023). Prediction of Risk of Frailty Among Institutionalized Older Adults in India. Current Aging Science, 16(1), 33–39. DOI: 10.2174/1874609815666220415130737.	PubMed/Scopus	Institutionalized older adults in residential care facilities	Document access restricted by publisher paywall with no institutional license available; repository link inactive; reprint request to authors unanswered.
UR-03	Ponvel, P., Murukesu, R.R., Shahar, S., Rivan, N.F.M., Subramaniam, P., & Singh, D.K.A. (2024). Transition of Physical, Psychological, and Cognitive Frailty and Its Associated Determinants in Malaysian Older Adults: A 5-Year Follow-up Study. Rejuvenation Research, pp. 198–206. DOI: 10.1089/rej.2024.0047 [not independently verified].	CINAHL (EBSCOhost)		Full text unavailable through institutional library subscriptions or interlibrary loan networks; no response from the corresponding author following electronic inquiry.

Note: These reports were sought for retrieval during the eligibility screening phase (Figure 1, PRISMA 2020 flow diagram) but could not be obtained in full text prior to the eligibility assessment deadline. Author names and bibliographic details were cross-checked against the original publisher records; the DOI for UR-03 could not be independently confirmed at the time of writing and should be verified against the publisher’s record before submission.
